# Effects of doxycycline post-exposure prophylaxis for prevention of sexually transmitted infections on gonorrhoea prevalence and antimicrobial resistance among men who have sex with men in the USA: a modelling study

**DOI:** 10.1016/S2666-5247(24)00168-X

**Published:** 2024-10-04

**Authors:** Emily Reichert, Yonatan H Grad

**Affiliations:** Department of Immunology and Infectious Diseases, Harvard T H Chan School of Public Health, Boston, MA, USA

## Abstract

**Background:**

Doxycycline post-exposure prophylaxis (PEP) has been shown to be efficacious for the prevention of bacterial sexually transmitted infections, but resistance implications for *Neisseria gonorrhoeae* remain unknown. We aimed to use a mathematical model to investigate the anticipated impact of doxycycline PEP on the burden of gonorrhoea and antimicrobial resistance dynamics in men who have sex with men (MSM) in the USA.

**Methods:**

Using a deterministic compartmental model, characterising gonorrhoea transmission in a US MSM population comprising three sexual activity groups defined by annual partner turnover rates, we introduced doxycycline PEP at various uptake levels (10–90%) among those with high sexual activity. Infections were stratified by symptom status and resistance profile (ie, susceptible, ceftriaxone-resistant, tetracycline-resistant, or dual-resistant), with ceftriaxone the treatment for active infection. As resistance to tetracycline, not doxycycline, is monitored and reported nationally, we used this as a proxy for doxycycline PEP resistance. We compared the 20-year prevalence, incidence rates, and cumulative incidence of gonococcal infection, resistance dynamics (time to 5% prevalence of ceftriaxone resistance, 5% prevalence of dual resistance, and 84% prevalence of tetracycline resistance), and antibiotic consumption with baseline (ie, no doxycycline PEP).

**Findings:**

Uptake of doxycycline PEP resulted in substantial reductions in the prevalence and incidence of gonorrhoea, but accelerated the spread of tetracycline resistance. The maximum reduction in prevalence over 20 years compared with no uptake ranged from 40·3% (IQR 15·3–83·4) with 10% doxycycline PEP uptake to 77·4% (68·4–84·9) with 90% uptake. Similarly, the maximum reduction in the incidence rate ranged from 38·6% (14·1–83·6) with 10% uptake to 77·6% (68·1–84·7) with 90% uptake. Cumulative gonococcal infections were reduced by a median of 14·5% (IQR 8·4–21·6) with 10% uptake and up to 46·2% (26·5–59·9) with 90% uptake after 5 years, and by 6·5% (3·4-13·0) with 10% uptake and 8·7% (4·3–36·2) with 90% uptake by 20 years. In almost all scenarios explored, doxycycline PEP lost clinical effectiveness (defined as 84% prevalence of tetracycline resistance) within the 20-year period, but its lifespan ranged from a median of 12·1 years (IQR 9·9–15·7) with 10% uptake to 1·6 years (1·3–1·9) with 90% uptake. Doxycycline PEP implementation had minimal impact on extending the clinical lifespan of ceftriaxone monotherapy (5·0 years [IQR 4·0–6·2]), with the median time to 5% prevalence of resistance ranging from 4·8 years (3·9–6·0) for 90% uptake to 5·0 years (4·1–6·2) for 10% uptake. Similarly, the median time to 5% prevalence of dual resistance to ceftriaxone and tetracycline ranged from 4·8 years (3·9–6·0) for 90% uptake to 5·8 years (4·8–7·4) for 10% uptake. Median decrease in ceftriaxone consumption for high doxycycline PEP uptake levels compared with baseline ranged from 41·7% (27·0–54·3) for 50% uptake to 50·2% (29·3–62·7) for 90% uptake at 5 years, but dropped to 11·8% (6·9–32·0) for 50% uptake and 12·1% (7·0–41·6) for 90% uptake after 20 years.

**Interpretation:**

Notwithstanding the clear benefits of doxycycline PEP for other sexually transmitted infections, for *N gonorrhoeae*, model findings suggest that doxycycline PEP is an effective but impermanent solution for reducing infection burden, given eventual selection for resistant strains. This finding presents a challenge for policy makers considering strategies for doxycycline PEP implementation and oversight: the need to balance the clear, short-term clinical benefits with the risk of harm via antimicrobial resistance.

**Funding:**

US Centers for Disease Control and Prevention, National Institute of Allergy and Infectious Diseases.

## Introduction

Gonorrhoea, a highly prevalent sexually transmitted infection caused by *Neisseria gonorrhoeae* (the gonococcus), has a decades-long record of antibiotic evasion, and only ceftriaxone remains recommended for its treatment in the USA.^1^ The scarcity of widely effective antibiotics for the treatment of gonorrhoea underscores the need for other tools, such as vaccines and prophylactic therapies, to control the burden of infection.

Doxycycline post-exposure prophylaxis (PEP), a 200 mg dose of the broad-spectrum antibiotic doxycycline within 72 h after sexual contact, has shown evidence in clinical trials conducted in men who have sex with men (MSM) of reducing the incidence of bacterial sexually transmitted infections in populations at high risk.^2-4^ Whereas risk reductions have been consistent for chlamydia and syphilis across studies in MSM, results for gonorrhoea have been mixed, and a trial in cisgender women in Kenya did not show benefit, with low adherence as one possible explanation for this outcome.^2-5^ One hypothesis attributes varying efficacy estimates for doxycycline PEP in preventing gonococcal infection to differences in the prevalence of resistance, usually measured as resistance to tetracycline, an antibiotic of the same class. Resistance to tetracyclines can be plasmid-encoded via *tetM*, conferring high-level resistance, and chromosomally encoded, with mutations in the MtrCDE efflux pump and its transcriptional repressor MtrR, porins, and the RpsJ Val57Met target combining to confer low-level resistance.^6^ However, although tetracycline resistance, measured in terms of minimum inhibitory concentration (MIC), correlates with doxycycline MIC,^7^ it is not clear how MIC relates to the efficacy of doxycycline as a prophylactic rather than as treatment.

Following a series of recommendations from state and local health departments, the US Centers for Disease Control and Prevention (CDC) proposed national guidelines for the use of doxycycline PEP for bacterial sexually transmitted infections. These guidelines recommend consideration of doxycycline PEP for MSM and transgender women with a history of at least one bacterial sexually transmitted infection in the past 12 months.^8^ By contrast, the UK Health Security Agency cited antimicrobial resistance concerns as a reason not to endorse doxycycline PEP.^9^ The proposed CDC guidelines acknowledge concerns around antimicrobial resistance and the scarcity of studies evaluating the effect of doxycycline PEP, suggesting that “potential risks related to the development of resistance… will need to be closely monitored after implementation”.^8^

To bolster evidence of the effect of doxycycline PEP on antimicrobial resistance in *N gonorrhoeae* and to help inform monitoring efforts, we aimed to explore the effect of doxycycline PEP implementation on gonococcal infection and resistance dynamics in a large population through a mathematical model of gonorrhoea transmission among MSM.

## Methods

### Study design

We adapted a deterministic compartmental model characterising gonorrhoea transmission in a population representative of MSM in the USA.^10^ We added an exposure compartment to study the dynamics of administering doxycycline PEP to a proportion of exposed individuals (ξ_B_), transforming the model into a susceptible–exposed–infectious–susceptible model (table 1, appendix p 2). For this study, exposure was defined by a partnership resulting in a gonococcal infection transmission event to a susceptible individual. Individuals spent on average 1 day in the exposure (or latent) compartment, after which they either progressed to infection or transitioned back to susceptibility following successful doxycycline PEP. The rate of removal from the exposed compartment (γ=1/[1 day]) aligned with the recommended 72 h window for doxycycline PEP after exposure (NCT03980223).^8^

In brief, the model characterised an MSM population (N=10^6^) stratified into three sexual activity groups (low, intermediate, and high) characterised by annual rates of partner change (θ), with individuals of different sexual activity groups interacting with mixing parameter ε. Individuals aged into and out of the sexually active population at rate ρ, contributing for 20 years on average. Individuals with infection could recover spontaneously or through antibiotic treatment with ceftriaxone monotherapy. Infections were stratified by symptom status and resistance profile, where each infection could be resistant to ceftriaxone, tetracycline, neither, or both. Because resistance to tetracycline, not doxycycline, is monitored and reported in the USA, we used this as a proxy for resistance to doxycycline and conservatively assumed that only high-level tetracycline resistance (MIC >8 μg/mL) renders doxycycline PEP ineffective (appendix p 19).^6^

Details on the model structure, parameterisation, equations, and sensitivity analyses are in the appendix (pp 17-24). No ethics approval was required for this modelling study.

### Procedures

We ran the model under baseline parameterisation (table 1) over 20 years using R package deSolve^19^ to observe projected infection and resistance dynamics following doxycycline PEP implementation at time t=0. A range of potential uptake, or utilisation, levels were explored (0%, 10%, 25%, 50%, 75%, and 90%) that characterised the proportion of gonorrhoea-exposed individuals within the high sexual activity group administered doxycycline PEP as intended. The high sexual activity group constituted a fixed 10% of the population, and doxycycline PEP use was restricted to this group in line with current US policies recommending doxycycline PEP only for individuals at high risk of infection. This approach is not concordant with, but only an approximation of, the high-risk definition used in clinical trials and CDC guidelines, which require one or more bacterial sexually transmitted infection diagnosis within the past 12 months.^3,4,8^ Doxycycline PEP use in the low and intermediate sexual activity groups (comprising 90% of the population) was fixed at 0%.

### Outcomes

We evaluated multiple primary outcomes over 20 years following the introduction of doxycycline PEP, including: the prevalence, incidence rate, and cumulative number of gonococcal infections; the cumulative number of ceftriaxone treatments administered; and the time until 5% resistance prevalence for ceftriaxone, 5% resistance prevalence for dual resistance, and 84% high-level resistance prevalence for tetracycline. The 5% resistance prevalence threshold for ceftriaxone constitutes the WHO threshold for revisiting treatment guidelines.^20^ For tetracycline, because the estimated high-level resistance in the US MSM population (10–9%) is above 5% prevalence at baseline,^6^ we arrived at 84% prevalence by calculating the threshold for which the risk of infection with doxycycline PEP use was reduced by 10% or less (appendix p 22). We associate this endpoint with loss of clinical utility of doxycycline PEP, assuming it would no longer be a desirable prevention measure.

The 20-year prevalence and incidence rate trajectories of gonococcal infection by uptake of doxycycline PEP were illustrated visually only for the baseline model parameterisation scenario (table 1). However, to account for parameter uncertainty, 1000 iterations of the model were run, parameterised using random draws from probability distributions for select parameters (table 1; appendix p 3). Quantitative model outcomes were summarised using medians (IQRs).

### Sensitivity analysis

To explore the generalisability of the results to settings with different starting levels of tetracycline resistance, we reran the model varying this parameter (r_B_=0·05, 0·25, 0·50, or 0·75) in a univariate sensitivity analysis. Results also inform infection and resistance dynamics if the assumption that only high-level tetracycline resistant strains (MIC >8 μg/mL) confer resistance to doxycycline PEP was incorrect, and a greater proportion of infections are immune to doxycycline PEP at baseline. The r_B_=0·25 scenario approximates model expectations if any tetracycline resistance (MIC ≥2 μg/mL), estimated at 26·8% prevalence in US MSM, confers resistance to doxycycline PEP.^6^

Next, we conducted two analyses reflecting different doxycycline PEP roll-out strategies for comparison. The first strategy kept use of doxycycline PEP restricted to the high sexual activity group but complemented the intervention with accelerated sexually transmitted infection screening for gonococcal infection, per the CDC recommendation.^8^ The screening rate among the high sexual activity group was increased as a function of doxycycline PEP uptake, holding constant the baseline screening rate (T_m_=0·36) in the remaining 90% of the population. The second strategy presented non-targeted doxycycline PEP roll-out, expanding access to all sexual activity groups. This universal implementation approach assumed use of doxycycline PEP was equivalent for all individuals, independent of risk (appendix pp 22-23).

Finally, to explore the sensitivity of model outcomes to key parameters of interest, we conducted two supplementary analyses. The risk ratio parameter κ, measuring the effectiveness of doxycycline PEP in preventing infection to strains without high-level resistance, captures the effectiveness of doxycycline PEP against susceptible, intermediate, or low-level resistant strains (tetracycline MIC ≤8 μg/mL) in a real-world setting, accounting for factors such as medication adherence. To evaluate the impact of doxycycline PEP effectiveness (1 – κ), we varied it from 20% to 100% and again assessed 20-year trends in prevalence of infection under a range of uptake scenarios. Then, to evaluate the influence of assumptions about tetracycline resistance, we conducted a bivariate sensitivity analysis for the fitness cost associated with high-level tetracycline resistance (1–f_B_: 0–0·20) and the probability of de novo resistance emergence (ω_B_: 0–10^−4^) when doxycycline PEP is used. All analyses were run using R version 4.1.2. All code needed to run the model, analyse data, or visualise results is available at https://github.com/emreichert13/doxypep.

### Role of the funding source

The funder of the study had no role in study design, data collection, data analysis, data interpretation, or writing of the report.

## Results

**Figure 1:**
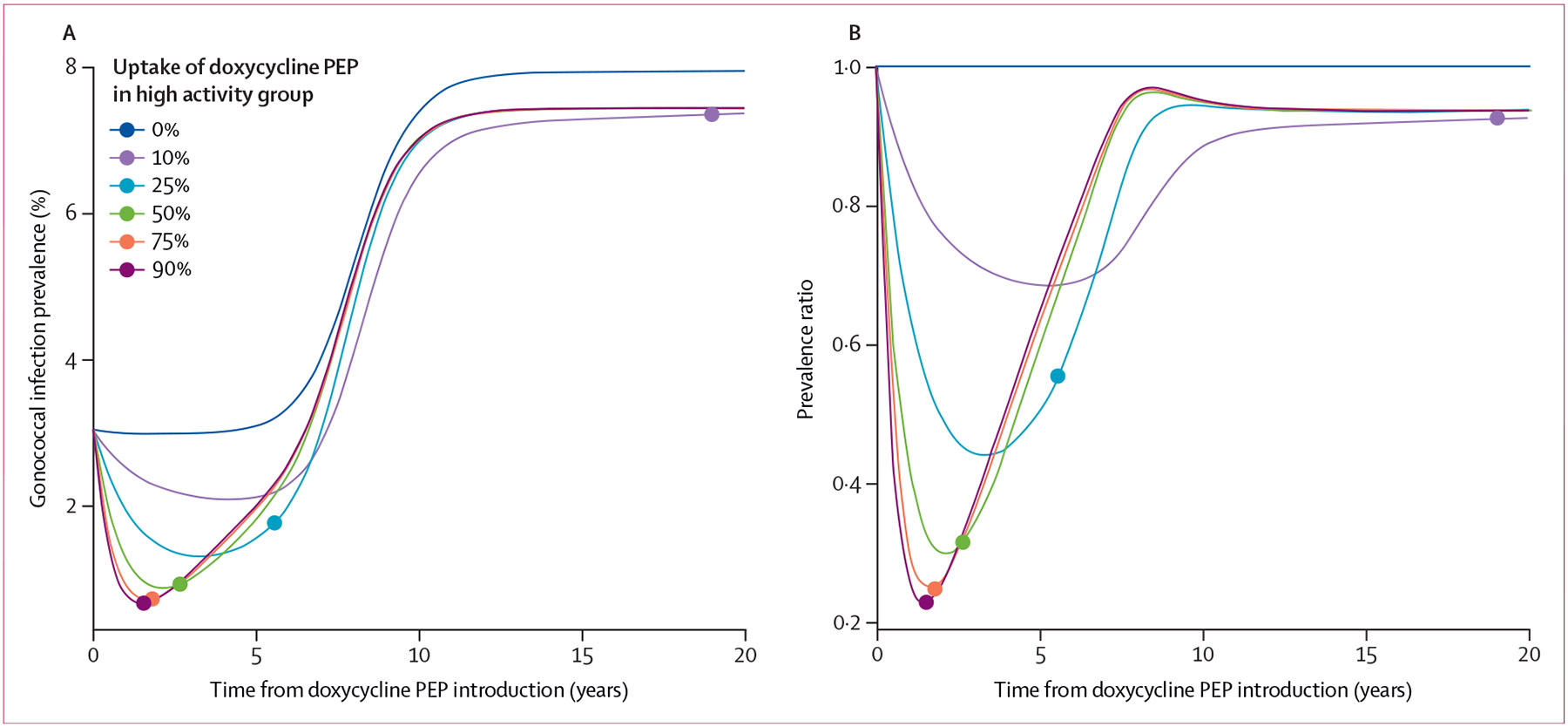
Prevalence of gonococcal infection over time for varying levels of doxycycline PEP uptake (A) Prevalence at each timepoint calculated as the total number of infections over the total population size (N=10^6^). (B) Prevalence ratio, where results were normalised (or divided by) the prevalence under the scenario with no introduction of doxycycline PEP (0% uptake). Doxycycline PEP uptake was defined as the proportion of exposed individuals treated with doxycycline PEP within the high sexual activity population. The dot on each line represents the time at which high-level tetracycline resistance (which we assume confers doxycycline PEP resistance) reached 84% prevalence under that uptake level, the threshold at which there was 10% or less reduction in the risk of infection with doxycycline PEP use that is associated with the loss of clinical utility. PEP=post-exposure prophylaxis.

**Figure 2:**
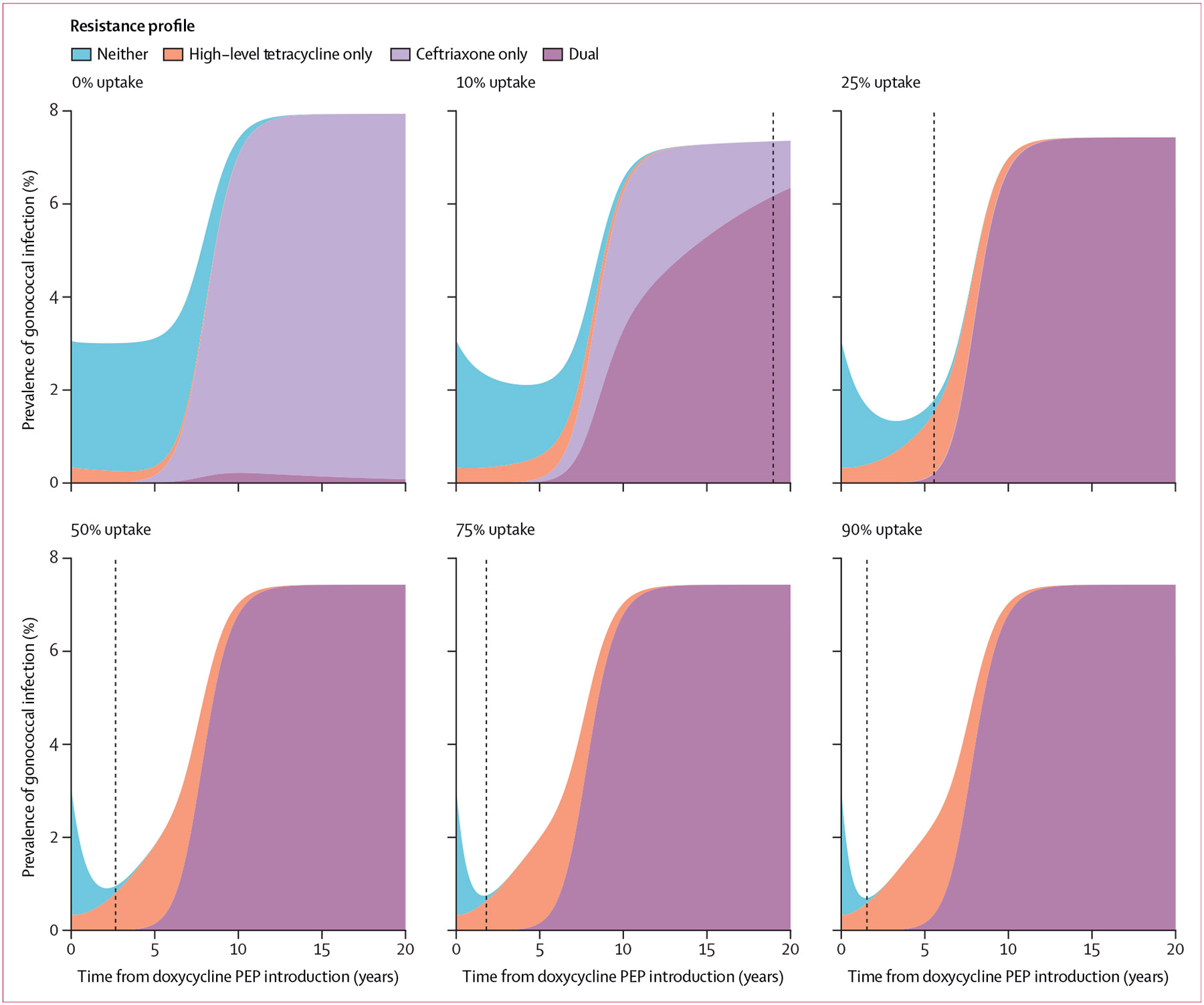
Prevalence of gonococcal infection by resistance profile over time, by proportion of doxycycline PEP uptake High-level tetracycline resistance (minimum inhibitory concentration >8 μg/mL) is assumed to confer resistance to doxycycline PEP. Doxycycline PEP uptake was defined as the proportion of exposed individuals treated with doxycycline PEP within the high sexual activity population. Black dashed lines indicate the time at which the 84% tetracycline resistance threshold is met, assumed to warrant discontinuation of doxycycline PEP due to widespread high-level tetracycline resistance and loss of clinical utility. PEP=post-exposure prophylaxis.

Under baseline parameterisation, with no introduction of doxycycline PEP, the prevalence of gonococcal infection remained stable at approximately 3% over 5 years (figures 1-2). Then, triggered by an increase and eventual takeover of ceftriaxone resistance, which met the 5% prevalence threshold at 5·0 years, gonococcal infection prevalence increased and re-equilibrated to approximately 8% (figures 1-2).

Implementing doxycycline PEP at any uptake level (≥10%) in the high sexual activity group substantially reduced the prevalence of gonococcal infection over the initial implementation period (figures 1-2). Following the introduction of doxycycline PEP, the prevalence of infection at its lowest point was reduced by a median of 77·4% (IQR 68·4–84·9) with 90% uptake, with the magnitude of the maximum prevalence reduction increasing with doxycycline PEP uptake level (table 2). Incidence rates for gonorrhoea showed highly similar trends to prevalence, with maximum reductions in the incidence rate ranging from 38·6% (14·1–83–6) with 10% uptake to 77·6% (68·1–84·7) with 90% uptake compared with baseline (appendix p 4). Cumulative gonococcal infections after 5 years were reduced by a median of 14·5% (8·4–21·6) with 10% doxycycline PEP uptake and up to 46·2% (26·5–59·9) with 90% uptake, relative to the ceftriaxone monotherapy status quo (table 2). As time since doxy-PEP introduction increased, and high-level tetracycline resistance became increasingly widespread, the benefit of doxycycline PEP tapered. After 20 years, differences in gonococcal infection prevalence across doxycycline PEP uptake levels (≥10%) had largely disappeared, evidenced by 0% median prevalence reductions relative to baseline (table 2). By year 20, the median reduction in cumulative infections was 6·5% (3·4–13·0) with 10% uptake and 8·7% (4·3–36·2) with 90% uptake, compared with no doxycycline PEP use (table 2).

As the use of doxycycline PEP increased, the median time until high-level tetracycline resistance met the 84% prevalence threshold (ie, when doxycycline PEP lost clinical utility) decreased, from a median of 12·1 years (IQR 9·9–15·7) with 10% uptake to 1·6 years (1·3–1·9) with 90% uptake (figure 2, table 2). Across uptake levels, the implementation of doxycycline PEP did not substantively affect the time until ceftriaxone resistance or dual resistance met 5% prevalence, as both remained at a median of approximately 5 years (table 2). Median ceftriaxone consumption at 5 years was more than 40% lower for high doxycycline PEP uptake levels (≥50%) compared with baseline; after 20 years, this difference had narrowed to approximately 12% (table 2).

**Figure 3:**
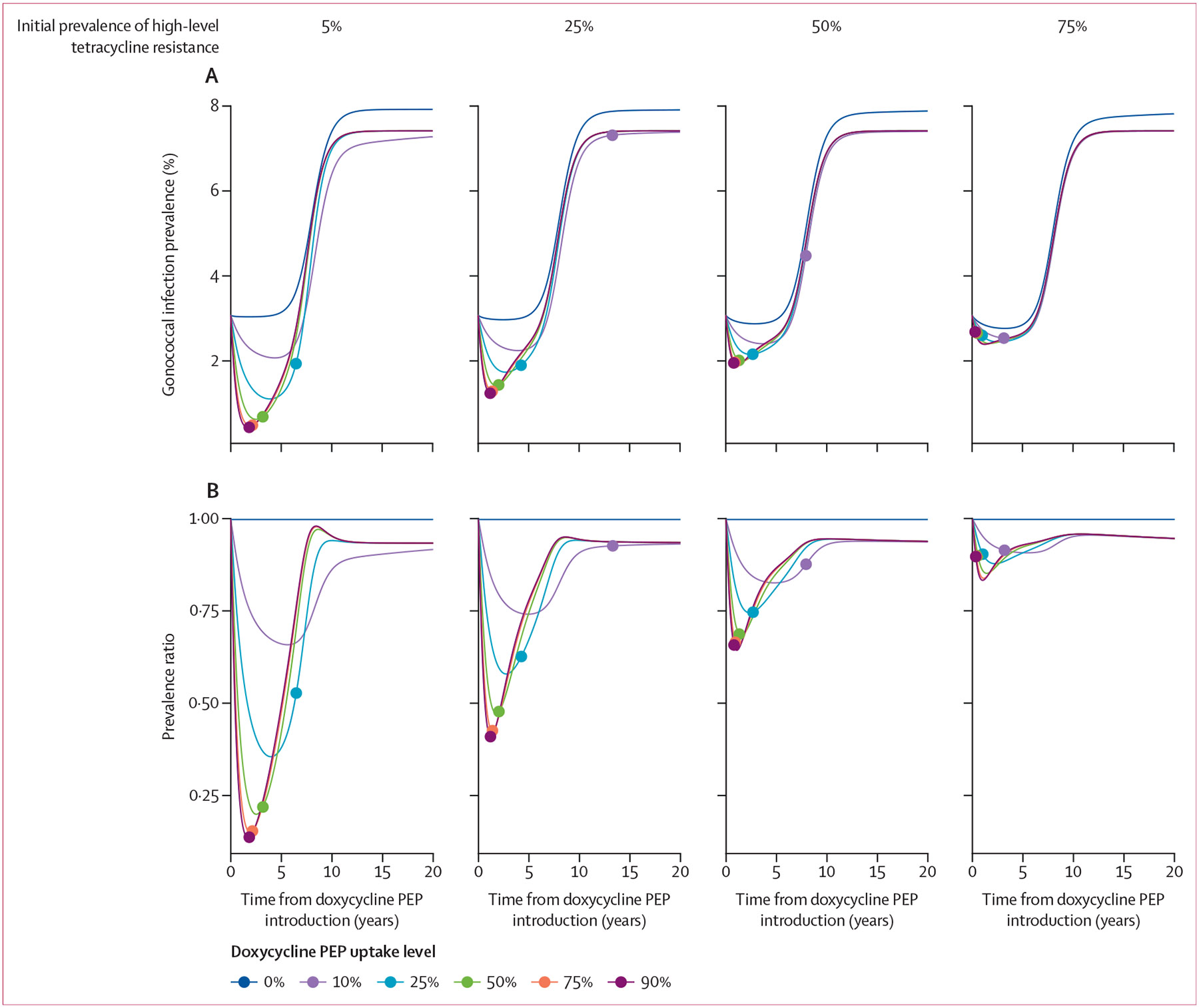
Prevalence of gonococcal infection over time for varying doxycycline PEP uptake levels, by the prevalence of high-level tetracycline resistance at time 0 (A) Absolute prevalence estimates over time, calculated as the total number of gonococcal infections over the total population size (N=10^6^) under each doxycycline PEP use scenario. (B) Prevalence ratio estimates over time, where results are normalised (or divided by) the prevalence under the scenario with no doxycycline PEP introduction (0% uptake). Initial prevalence of high-level tetracycline resistance is the prevalence at the start of the model, ranging from 5% to 75%. Doxycycline PEP uptake is defined as the proportion of exposed individuals treated with doxycycline PEP within the high sexual activity population. The dot on each line represents the time at which high-level tetracycline resistance reached 84% prevalence under that uptake level, the threshold at which there was 10% or less reduction in risk of infection with doxycycline PEP use that is associated with the loss of clinical utility. Under baseline model parameterisation, the prevalence of high-level tetracycline resistance is 10·9%. PEP=post-exposure prophylaxis.

In the sensitivity analyses, greater prevalence of high-level tetracycline resistance (or resistance to doxycycline PEP) at the model start accelerated the time until loss of clinical utility of doxycycline PEP (>84% prevalence of resistance) and attenuated the benefit of doxycycline PEP in reducing the gonococcal infection burden (figure 3; appendix p 5). Assuming only 5% of strains circulating at baseline are doxycycline PEP resistant allows 90% doxycycline PEP uptake to reduce infections by a median of 53·8% (IQR 33·5–68·7) over 5 years; by contrast, with 75% resistance prevalence at baseline, this reduction is 8·3% (3·4–12·6).

Supplementing doxycycline PEP with enhanced screening for gonococcal infection was estimated to be highly effective in reducing gonorrhoea prevalence within the 5 years after implementation (appendix pp 8-10). Prevalence of infection was reduced by a median of 98% or more with uptake of 50% or more, and there was a reduction in cumulative infections at 5 years after implementation of 28·4% (IQR 17·7–40·2) with 10% doxycycline PEP uptake and 74·5% (54·5–86·0) with 90% uptake, relative to the ceftriaxone monotherapy status quo. Due to the spread and eventual takeover of both ceftriaxone and high-level tetracycline resistance, infections rebounded within the 20-year window under all doxycycline PEP uptake scenarios explored, with a median prevalence reduction at 20 years of 0% across uptake levels. The clinical lifespan of doxycycline PEP was not extended relative to the primary analysis, and that of ceftriaxone was shortened with high doxycycline PEP and screening levels, ranging from 4·0 years (IQR 3·4–4·8) with 10% uptake to 1·9 years (1·7–2·1) with 90% uptake.

Expanding doxycycline PEP access to all individuals in the model population, regardless of sexual activity group, led to 20-year prevalence trends highly similar to the primary analysis (appendix pp 11-13). At 5 years, cumulative infections were reduced by a median of 17·3% (IQR 10·6–26·0) under the 10% doxycycline PEP uptake scenario to 49·4% (27·5–63·0) with 90% doxycycline PEP uptake, relative to no doxycycline PEP use. The time to loss of clinical utility of doxycycline PEP was reduced compared with the primary analysis at low uptake levels, decreasing from a median of 12·1 years (IQR 9·9–15·7) to 9·5 years (8·0–11·9) under 10% doxycycline PEP uptake (table 2; appendix pp 12-13). Other quantitative outcomes remained similar to the primary analysis, even though across uptake levels, median absolute consumption of doxycycline PEP increased by 102–112% (5 years) to 149–153% (20 years) under this universal implementation approach relative to targeted implementation.

Finally, under baseline parameterisation, model outcomes showed varying levels of sensitivity to key parameters of interest in univariate and bivariate sensitivity analyses. Increases in the risk ratio of gonococcal infection per doxycycline PEP use parameter (κ: 0–0·8), corresponding to decreased effectiveness of doxycycline PEP, limited the ability of doxycycline PEP to control the burden of infection, but time until loss of its clinical utility was extended (appendix p 14). For 50% doxycycline PEP uptake with κ=0, cumulative infections were 56·8% lower at 5 years relative to no doxycycline PEP uptake, but the 84% resistance prevalence threshold was met at 1·7 years. By contrast, for κ=0·8, cumulative infections were 33·4% lower at 5 years and the resistance threshold was met at 8·5 years.

Model outcomes were insensitive to the parameter characterising probability of de novo resistance emergence (ω_b_: 0–10^−4^) with doxycycline PEP use over the explored range. By contrast, variation in the relative fitness of high-level tetracycline-resistant strains (f_B_) led to qualitatively different gonococcal infection and resistance dynamics (appendix pp 15-16). Increasing the fitness cost extended the time until the loss of clinical utility of doxycycline PEP and substantially reduced the 20-year burden of gonococcal infection, particularly with high doxycycline PEP uptake. With a 20% fitness cost (f_B_=0·80) and a doxycycline PEP uptake level of 50% or more, the 20-year cumulative number of infections was reduced by 92·9% or more relative to no doxycycline PEP.

## Discussion

Our analysis showed that under most model parameterisations, doxycycline PEP implementation was an effective albeit temporary intervention for reducing the burden of gonorrhoea in a US MSM-like population. Doxycycline PEP use corresponded to large initial reductions in gonorrhoea prevalence and incidence; however, increasing doxycycline PEP use also accelerated the loss of its clinical utility, with the direct prophylactic benefit of doxycycline PEP almost always lost within 20 years. This effect was not due to the evolution of resistance on doxycycline PEP treatment: model outcomes were insensitive to this parameter, potentially because substantial high-level resistance at baseline (10·9%) renders rare de novo resistance emergence events inconsequential. Rather, observed resistance dynamics resulted from doxycycline PEP’s population-level prevention of infections caused by susceptible strains and preferential selection for strains with pre-existing resistance. This result is in keeping with the anticipated consequence of a trial of minocycline PEP in heterosexual men, which selected for resistant *N gonorrhoeae*.^21^

Notably, the introduction of doxycycline PEP into the model population did not buy more time in terms of the clinical lifespan of ceftriaxone. Across doxycycline PEP implementation strategies and uptake levels (0–90%), the time until 5% ceftriaxone resistance prevalence was met, warranting new therapeutics, stayed relatively constant at a median of 5 years (table 2; appendix pp 12-13) or decreased with accelerated screening (appendix pp 9-10). Of note, this measure only reflects the proportion of resistant infections (not the absolute number).

A dual intervention pairing doxycycline PEP uptake with increased sexually transmitted infection screening for individuals in the high sexual activity group, as is recommended in most doxycycline PEP guidelines to date, maintained very low prevalence of gonococcal infection for more than 5 years on average (appendix pp 8-10). Despite loss of clinical utility of doxycycline PEP in line with the primary analysis and a subsequent rebound in infections, the accelerated screening component was crucial to minimising the burden of gonococcal infection for a longer period compared with doxycycline PEP alone.

Expanding doxycycline PEP access to the entire model population increased absolute doxycycline consumption by 149–153% at 20 years across uptake scenarios, but showed little to no improvement in reducing the gonococcal infection burden, relative to the targeted approach. This evidence suggests current doxycycline PEP guidelines, with a focus on MSM at high risk for acquiring and transmitting sexually transmitted bacterial infections, are most effective in maximising the clinical benefit of doxycycline PEP while minimising its consumption. However, offering doxycycline PEP at high uptake levels more broadly showed no substantial acceleration of antimicrobial resistance in *N gonorrhoeae* relative to targeted implementation.

Model outcomes showed sensitivity to the fitness cost of tetracycline (ie, doxycycline PEP) resistance. High fitness costs paired with high doxycycline PEP uptake substantially reduced gonorrhoea prevalence over 20 years (appendix pp 15-16). Underlying resistance dynamics show that, although dual resistant strains still increase to comprise 99% or more of infections within 20 years with doxycycline PEP uptake of 50% or more, the drastically reduced fitness of dual resistant strains maintains the low gonococcal infection burden even after loss of doxycycline PEP effectiveness. However, as of 2018, the estimated prevalence of tetracycline resistance in the MSM population in the USA was substantial (26·8% resistant; 10·9% high-level resistant),^6^ and strains carrying *tetM* are widespread globally.^22,23^ This persistence of resistant strains in the absence of direct selective pressure suggests resistance might not incur a high fitness cost, as tetracyclines have not been recommended for gonorrhoea treatment since the 1980s in the USA and other countries.^23^

Interpretation of model projections warrants caution. We assumed, within each uptake level, that ceftriaxone and doxycycline PEP use remained constant over time. Beyond the point at which high-level tetracycline resistance reaches 84% prevalence, and doxycycline PEP is therefore less than 10% effective in preventing infection (assuming κ=0·38), it is unlikely that doxycycline PEP use would be maintained. Similarly, per WHO recommendations, once ceftriaxone resistance reaches 5% prevalence, treatment protocols require revision. Changes in treatment regimens could impact selective pressures and alter the prevalence of gonorrhoea the model re-equilibrates to following widespread doxycycline PEP, or ceftriaxone, failure. Future projections also rely on current gonorrhoea dynamics being maintained, but new tools for gonorrhoea management and prevention and changing sexual behaviours might disrupt dynamics within the next 20 years. We therefore emphasise that our modelled trajectories are not meant to accurately predict long-term gonococcal infection burden. We instead use results relatively, to compare various doxycycline PEP interventions with a status quo scenario with no disruptions to the way gonorrhoea is currently managed.

We did not attempt to model specific mechanisms of resistance but conservatively assumed resistance mechanisms to ceftriaxone and tetracycline were independent, despite one global study of *penA60*-harbouring ceftriaxone-resistant strains finding that 70·7% were tetracycline resistant, suggesting an association.^24^ The study and others have limited ability to examine this association in the USA, given the rarity of detected ceftriaxone resistance to-date.^6^ If ceftriaxone resistance is more likely to emerge in tetracycline resistant strains, or if failed doxycycline PEP followed by ceftriaxone treatment fosters an environment conducive to dual resistance emergence, loss of effectiveness of both ceftriaxone and doxycycline PEP would be accelerated.

We note that the true values for the rate of emergence and the fitness cost of ceftriaxone resistance are unclear and that the rate of increase in ceftriaxone resistance prevalence in our results exceeds what has been seen in the USA to date. Although surveillance of *N gonorrhoeae* ceftriaxone MICs shows that ceftriaxone resistance has been slow to emerge in the USA, it is difficult to discern the relationship between ceftriaxone use and the emergence and spread of ceftriaxone resistance from this observation. This difficulty is partly due to the changing recommended dose of ceftriaxone (increasing from 125 mg to 250 mg to 500 mg in the USA, given concern for emerging ceftriaxone resistance), the use of dual therapy of ceftriaxone with azithromycin from 2012 to 2020, and the likely variation in the relevant parameters, such as fitness cost of resistance, depending on the genomic backgrounds of circulating resistant strains. Furthermore, the low level of resistance to ceftriaxone in the USA contrasts with recent data from several countries in Asia, with regions reporting ceftriaxone resistance greater than 20–30%, thus suggesting that a rapid rise in the prevalence of ceftriaxone resistance can and does occur.^25-27^ Nonetheless, we underscore that the model output should be understood as a relative trend rather than as a quantitative forecast. Both a lower rate of ceftriaxone resistance emergence and a higher fitness cost of ceftriaxone resistance would each be expected to slow the increase in prevalence of ceftriaxone resistance and the prevalence of gonococcal infection across the scenarios we examined, but not to alter the relative trends across levels of doxycycline PEP uptake.

Using a theoretical, compartmental mathematical model to approximate the complex dynamics of sexual behaviour and gonorrhoea transmission forces many limiting assumptions.^10^ There is no heterogeneity in the modelled population except for sexual activity, defined by the annual rate of partner turnover, and sexual mixing assortativity. Even this heterogeneity is simplified into three discrete categories. Sexual partnerships with repeated exposure to infection are not represented. Infections are homogeneous beyond symptom status and resistance profile; the model does not differentiate by sexual behaviour, type of sexual contact, or anatomical site of infection. Model parameters thus represent a simplified average value, whereas the probability of symptomatic infection or transmission is likely to vary by factors such as the type of sexual contact and anatomical site. Although we account for parameter uncertainty, no model (structural) uncertainty is explored, presenting an opportunity for future work.

The model ignores the potential for bystander selection—ie, selection experiences by *N gonorrhoeae* attributable to treatment with ceftriaxone or tetracyclines for other, co-occurring indications. One study estimates that bystander experiences comprise 4·8% of *N gonorrhoeae*’s ceftriaxone exposures and 25–29·7% of doxycycline exposures, but how substantially these bystander experiences contribute to resistance is not well understood.^28,29^ The model also excludes importation of drug-resistant strains into the population and ignores any off-label antibiotic use.

Our results for doxycycline PEP and *N gonorrhoeae* underscore the tension between the near-term clinical benefit of disease prevention—including effectiveness in preventing syphilis and chlamydia—and the potential future harm of resistance, raising ethical issues similar to those seen with mass antibiotic administration.^30^ Guidance for the provision of doxycycline PEP must also take into account the target populations of this intervention—gay and bisexual MSM and transgender women, populations too-often subjected to discrimination and homophobia when seeking health-care services—when considering the nuanced implications of limiting prophylaxis, both in initial guidelines and as doxycycline PEP and other treatment and prevention tools reshape disease and microbial ecologies.^31^

Doxycycline PEP can achieve substantial reductions in gonorrhoea prevalence and incidence in the short-term, particularly when paired with accelerated screening for sexually transmitted infections. However, the effectiveness of doxycycline PEP for gonorrhoea prophylaxis is limited by pre-existing *N gonorrhoeae* resistance, and its sustainability is limited by selection for resistant strains. Moreover, doxycycline PEP does not appear to prolong the clinically useful lifespan of ceftriaxone monotherapy. Study findings highlight the need for enhanced surveillance and resistance monitoring with doxycycline PEP roll-out. Nonetheless, the clinical benefit of doxycycline PEP could be deployed to temporarily minimise the burden of infection and disease, buying time to develop and bring into practice new tools for gonorrhoea prevention and treatment.

## Supplementary Material

1

## Figures and Tables

**Table 1:** Model parameters

	Domain	Baseline value	Global uncertainty analysis		Sources and starting values, if fit via MLE procedure
			Probability distribution	Mean (95% CI)	
**Model calibration target**					
Gonorrhoea prevalence at start (calibration target mean)	(0–1)	0·03 (ie, 3·0%)	..	··	··
**Model population and sexual behaviour parameters**					
N: population size	(0–¥)	10^6^	Fixed	Fixed	Assumption
N_k_: size of sexual activity groups	··	··	Fixed	Fixed	Reichert et al,^10^ 2023, and Tuite et al,^11^ 2017
Low	(0–¥)	N_1_=3 × 10^5^	··	··	··
Intermediate	(0–¥)	N_2_=6 × 10^5^	··	··	··
High	(0–¥)	N_3_=1 × 10^5^	··	··	··
θ: rate of partner turnover per sexual activity group (per year)	··	··	Fixed	Fixed	Model fitting, starting values from Reichert et al,^10^ 2023
Low	(0–¥)	θ_1_=1 × 1·41	··	··	Starting value: θ_1_=1 × 1·22
Intermediate	(0–¥)	θ_2_=5 × 1·41	··	··	Starting value: θ_2_=5 × 1·22
High	(0–¥)	θ_3_=20 × 1·41	··	··	Starting value: θ_3_=20 × 1·22
ε: mixing parameter	(0–1)	0·25	Beta (4·4, 13·3)	0·25 (0·08 to 0·47)	Model fitting, starting value from Reichert et al,^10^ 2023:0·24
r_A_: proportion of cases ceftriaxone resistant at start	(0–1)	0·0001	Fixed	Fixed	CDC GISP^12^ 2020–21
r_B_: proportion of cases tetracycline resistant (high-level; MIC >8 μg/mL) at start	(0–1)	0·109 (0·05–0·75)	··	··	Mortimer and Grad^6^ 2023 (range)
r_AB_: proportion of cases dual resistant at start	(0–1)	r_A_ × r_B_	··	··	Assumption
ρ: model entry and exit rate (per year)	(0–¥)	1/20	Fixed	Fixed	Reichert et al,^10^ 2023
**Gonorrhoea natural history parameters**					
σ: proportion of incident infections that are symptomatic	(0–1)	0·45	Beta (10·7, 13·1)	0·45 (0·26 to 0·65)	Model fitting, starting value estimated from Barbee et al,^13^ 2014: 0·50
b: transmission probability per partnership	(0–1)	0·55	Beta (13·1, 10·7)	0·55 (0·35 to 0·74)	Model fitting, starting value estimated from Reichert et al,^10^ 2023 and Tuite et al,^14^ 2018: 0·50
1/δ: average time to natural recovery from infection (days)	(0–¥)	76	Gamma (25·9, 2·9)	76 (50 to 108)	Model fitting, starting value estimated from Barbee et al,^15^ 2021, and Barbee et al,^16^ 2022: 0·288 × 365
1/γ: average time exposed, in days (independent of doxycycline PEP treatment)	(0–¥)	1	Fixed	Fixed	Assumption
**Treatment parameters**					
1/T_s_: average time to screen and treat, symptomatic infection (days)	(0–¥)	15	Gamma (19·4, 0·8)	15 (9 to 23)	Model fitting, starting value estimated from Reichert et al,^10^ 2023: 15
1/T_sr_: average time to retreatment, symptomatic infection (days)	(0–¥)	(1/T_S_)×3	··	··	Reichert et al,^10^ 2023 and Tuite et al,^11^ 2017
1/T_m_: average time to screen and treat, asymptomatic infection (days)	(0–¥)	1022	Gamma (4642·2, 0·2)	1022 (993 to 1052)	Model fitting, starting value estimated from Reichert et al,^10^ 2023 and de Voux et al,^17^ 2019 2·5 × 365
ξ_B_: proportion of exposed individuals receiving doxycycline PEP (ie, uptake level)	(0–1)	0–0·90	··	··	Assumption, range explored in primary analysis
κ: proportion of doxycycline PEP treatments that do not prevent infection, for reasons not due to high-level tetracycline resistance	(0–1)	0·38 (0–0·80)	Beta (8·6, 14·0)	0·38 (0·20 to 0·58)	Assumption, estimated from Luetkemeyer et al,^4^ 2023 (range)
ω: probability of emergence of resistance upon treatment					
Ceftriaxone	(0–1)	ω_A_ = 10^−8^	Uniform (0, 2 × 10^−8^)	10^−8^ (5 × 10^−10^ to 1·95 × 10^−8^)	Tuite et al,^11^ 2017 and Vegvari et al,^18^ 2020
Tetracycline (high-level; MIC >8 μg/mL)	(0–1)	ω_B_ = 0	Uniform (0, 2 × 10^−8^)	10^−8^ (5 × 10^−10^ to 1·95 × 10^−8^)	Assumption (range)
f: relative fitness of resistant bacteria, compared with susceptible					
Ceftriaxone resistant	(0–1)	f_A_ = 0·98	Beta (0·9, 0·02)	0·98 (0·71 to 1)	Tuite et al,^11^ 2017
Tetracycline resistant (high-level; MIC >8 μg/mL)	(0–1)	f_B_ = 0·98 (0·80–1)	Beta (0·9, 0·02)	0·98 (0·71 to 1)	Assumption (range)
Dual resistance	(0–1)	f_AB_ = f_A_ × f_B_	··	··	Assumption
π_s_: probability of retreatment if initial treatment failure, symptomatic infection	(0–1)	0·90	Fixed	Fixed	Reichert et al,^10^ 2023 and Tuite et al,^11^ 2017

Baseline values for model parameters were derived from the literature, a combination of the literature (for a starting estimate) and the MLE model fitting procedure, and expert opinion and assumption when estimates were unavailable. For the global uncertainty analysis, proportions were modelled using beta distributions and average time to event outcomes using gamma distributions. The baseline value for each parameter was assigned to be the mean of the probability distribution. For all proportions, an SD of 0·10 was assumed. For average time to event parameters, a reasonable variance for the gamma probability distributions was determined using the literature or expert opinion and assumption. CDC=Centers for Disease Control and Prevention. GISP=Gonococcal Isolate Surveillance Project. MIC=minimum inhibitory concentration. MLE=maximum likelihood estimation. PEP=post-exposure prophylaxis.

**Table 2:** Clinically relevant endpoints by level of doxycycline PEP uptake in the high sexual activity group only

	0% uptake	10% uptake	25% uptake	50% uptake	75% uptake	90% uptake
**Time to key resistance thresholds, years**						
Ceftriaxone resistance, 5%	5·0 (4·0–6·2)	5·0 (4·1–6·2)	4·9 (4·0–6·1)	4·8 (3·9–6·0)	4·8 (3·9–6·0)	4·8 (3·9–6·0)
Dual resistance, 5%	7·5 (6·0–10·1)	5·8 (4·8–7·4)	5·1 (4·2–6·4)	4·8 (3·9–6·1)	4·8 (3·9–6·0)	4·8 (3·9–6·0)
High-level tetracycline resistance, 84%	Not observed*	12·1 (9·9–15·7)	5·3 (4·4–6·5)	2·7 (2·3–3·3)	1·9 (1·6–2·3)	1·6 (1·3–1·9)
**Maximum reduction over 20 years compared with no uptake of doxycycline PEP**						
Prevalence of gonococcal infection	NA	40·3% (15·3–83·4)	61·5% (34·9–85·5)	72·2% (55·4–85·1)	76·1% (65·0–84·9)	77·4% (68·4–84·9)
**Reduction in 5-year outcomes compared with no uptake of doxycycline PEP**						
Prevalence of gonococcal infection	NA	36·7% (10·8–58·9)	49·5% (10·0–80·8)	44·9% (2·3–84·8)	40·6% (0·5–84·4)	38·7% (0·3–84·1)
Cumulative gonococcal infections	NA	14·5% (8·4–21·6)	27·8% (17·0–40·1)	38·9% (23·3–51·9)	44·0% (25·7–57·4)	46·2% (26·5–59·9)
Cumulative ceftriaxone treatments	NA	15·9% (9·9–22·7)	30·3% (19·9–41·8)	41·7% (27·0–54·3)	48·0% (29·0–60·1)	50·2% (29·3–62·7)
**Reduction in 20-year outcomes compared with no uptake of doxycycline PEP**						
Prevalence of gonococcal infection	NA	0·4% (0·2–20·4)	0·0% (0·0–29·6)	0·0% (0·0–35·6)	0·0% (0·0–38·5)	0·0% (0·0–38·5)
Cumulative gonococcal infections	NA	6·5% (3·4–13·0)	8·3% (3·9–20·4)	8·6% (4·1–28·2)	8·7% (4·3–33·9)	8·7% (4·3–36·2)
Cumulative ceftriaxone treatments	NA	8·3% (5·1–14·5)	11·1% (6·6–23·2)	11·8% (6·9–32·0)	12·0% (6·9–38·8)	12·1% (7·0–41·6)

Data are median (IQR) and are shown for 1000 simulations, with parameter values drawn randomly from their distributions for model parameters included in the global sensitivity analysis in table 1. The time until high-level tetracycline resistance at 84% prevalence was used to approximate time to loss of the clinical usefulness of doxycycline PEP, whereas the 5% prevalence threshold for ceftriaxone resistance indicates when new therapeutics would be needed. NA=not applicable. PEP=post-exposure prophylaxis. *Not observed within the 20-year period over which the model was run.

## Data Availability

All code needed to simulate the data, run analyses, and visualise results are available at https://github.com/emreichert13/doxypep.

## Research in context

### Evidence before this study

Doxycycline post-exposure prophylaxis (PEP) reduces the incidence of syphilis, chlamydia, and in some contexts gonorrhoea in men who have sex with men (MSM) and transgender women.

We searched PubMed for modelling studies published between database inception and Jan 31, 2024, that evaluated the effect of antibiotics for PEP, using a combination of key search terms (“sexually transmitted diseases”, “sexually transmitted infections”, or “gonorrhea” and “prophylaxis” or “post-exposure prophylaxis”) plus “modeling”, with no language restrictions. We found studies of PEP, but none used modelling to quantitatively evaluate the effect of treatment on disease prevalence and antibiotic resistance.

### Added value of this study

Our modelling study evaluated the effect of the uptake of doxycycline PEP on gonorrhoea prevalence and resistance in MSM in the USA under a variety of implementation scenarios, adding to the existing knowledge on the impact of doxy-PEP on *Neisseria gonorrhoeae*.

### Implications of all the available evidence

Our findings support the need for enhanced surveillance and resistance monitoring of *N gonorrhoeae* following implementation of doxycycline PEP. Further modelling and clinical studies are warranted to bolster our understanding of the effect of doxycycline PEP on infection and resistance dynamics.
