# Supplementary material for: Effects of doxycycline post-exposure prophylaxis for prevention of sexually transmitted infections on gonorrhoea prevalence and antimicrobial resistance among men who have sex with men in the USA: a modelling study
Source: Lancet Microbe. Author manuscript; Available in PMC 2024 Dec 12. (PMC11635662; doi:10.1016/S2666-5247(24)00168-X)
Supplement: 1 [file NIHMS2034708-supplement-1.pdf]

THE LANCET
Microbe

Supplementary appendix

This appendix formed part of the original submission and has been peer reviewed.
We post it as supplied by the authors.

Supplement to: Reichert E, Grad YH. Effects of doxycycline post-exposure prophylaxis for prevention of sexually transmitted infections on gonorrhoea prevalence and antimicrobial resistance among men who have sex with men in the USA: a modelling study. *Lancet Microbe* 2024. https://doi.org/10.1016/S2666-5247(24)00168-X

Supplementary Material

Supplementary Figure 1. Schematic of gonorrhea transmission model. 2

Supplementary Figure 2. Probability density functions for parameters included in global sensitivity analysis. 3

Supplementary Figure 3. Incidence rate of gonococcal infection over time for varying doxy-PEP uptake levels (%). 4

Supplementary Table 1. Clinically relevant endpoints by level of doxy-PEP uptake (%) and prevalence of high-level tetracycline resistance at time 0. 5

Supplementary Figure 4. Prevalence of gonococcal infection over time for varying doxy-PEP uptake levels (%) and STI screening rates. 8

Supplementary Table 2. Clinically relevant endpoints by level of doxy-PEP uptake (%) and STI screening rate. 9

Supplementary Figure 5. Prevalence of gonococcal infection over time for varying doxy-PEP uptake levels (%) in the entire population. 11

Supplementary Table 3. Clinically relevant endpoints by level of doxy-PEP uptake (%) in the entire population. 12

Supplementary Figure 6. Prevalence of gonococcal infection over time for varying doxy-PEP uptake levels (%), by the per exposure risk ratio of infection for doxy-PEP users relative to non-users exposed to doxy-PEP susceptible strains. 14

Supplementary Figure 7. Prevalence of gonococcal infection over time for varying doxy-PEP uptake levels (%), by properties of doxy-PEP. 15

Supplementary Figure 8. Prevalence ratio of gonococcal infection over time for varying doxy-PEP uptake levels (%) relative to no doxy-PEP uptake, by properties of doxy-PEP. 16

Technical Supplement 17

Supplementary Tables and Figures

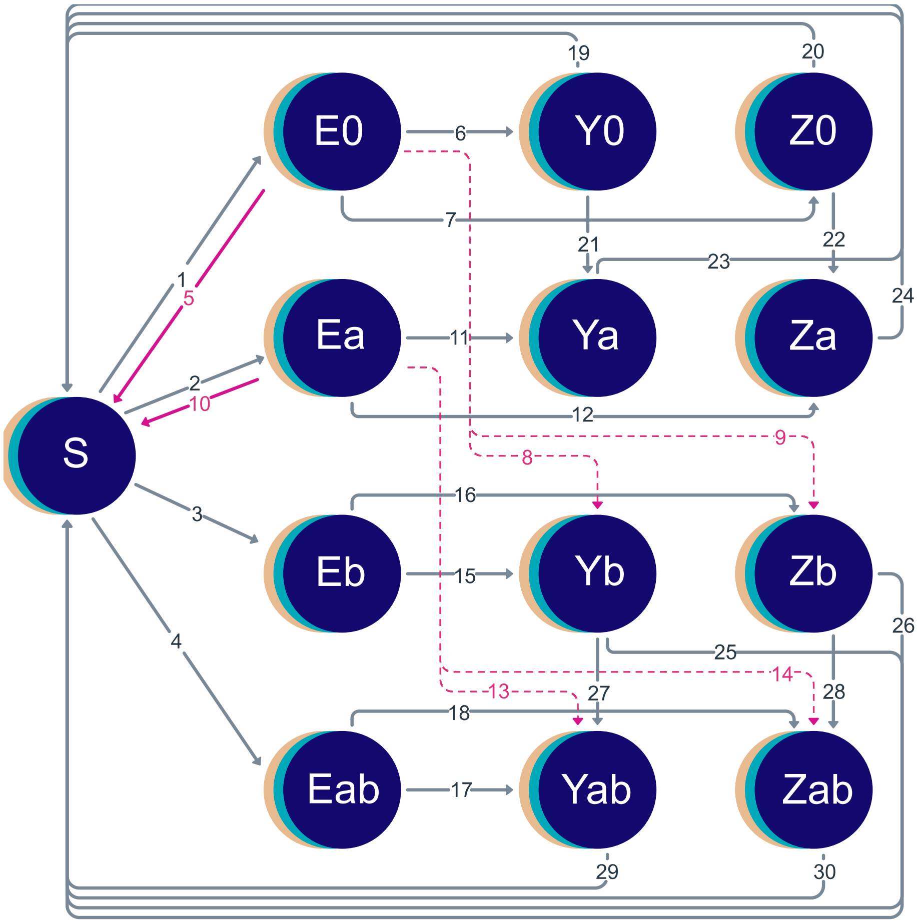

1	$\boldsymbol{\beta}\ (\mathbf{Y}_0 + \mathbf{Z}_0)$
2	$f_A\boldsymbol{\beta}\ (\mathbf{Y}_A + \mathbf{Z}_A)$
3	$f_B\boldsymbol{\beta}\ (\mathbf{Y}_B + \mathbf{Z}_B)$
4	$f_{AB}\boldsymbol{\beta}\ (\mathbf{Y}_{AB} + \mathbf{Z}_{AB})$
5, 10	$\xi_B\ (1-\kappa)\ (1-\omega_B)\ \gamma$
6, 11	$\sigma\gamma\ [(1-\xi_B) + \xi_B\ \kappa\ (1-\omega_B)]$
7, 12	$(1-\sigma)\gamma\ [(1-\xi_B) + \xi_B\ \kappa\ (1-\omega_B)]$
8, 13	$\sigma\xi_B\ \omega_B\ \gamma$
9, 14	$(1-\sigma)\xi_B\ \omega_B\ \gamma$
15, 17	$\sigma\gamma$
16, 18	$(1-\sigma)\gamma$
19, 25	$(1-\omega_A)\ T_s + \delta$
20, 26	$(1-\omega_A)\ T_m + \delta$
21, 27	$\omega_A T_s$
22, 28	$\omega_A T_m$
23, 29	$\pi_s T_{sr} + \delta$
24, 30	δ

Supplementary Figure 1. Schematic of gonorrhea transmission model. Abbreviations: S = Susceptible, E = Exposed, Y = Symptomatic Infection, Z = Asymptomatic Infection. Infections are further stratified by resistance profile, where 0 = susceptible, a = ceftriaxone resistant, b = high-level tetracycline resistant (minimum inhibitory concentration [MIC] >8 µg/mL), and ab = dual resistant. Overlapping discs for all compartments represent the model's stratification into three sexual activity groups: low, intermediate, and high. Arrows depict rates between compartments and would be multiplied by the compartment from which they flow to generate the model's set of differential equations (see Technical Supplement, p. 13-14). Arrows highlighted in pink represent transitions only possible with doxy-PEP implementation, and those that are dotted only occur if there is some non-zero probability of *de novo* resistance emerging with doxy-PEP use. Individuals can also enter and exit the population at rate ρ (arrows not shown). Definitions of all parameters used in rate equations can be found in Table 1 of the main text.

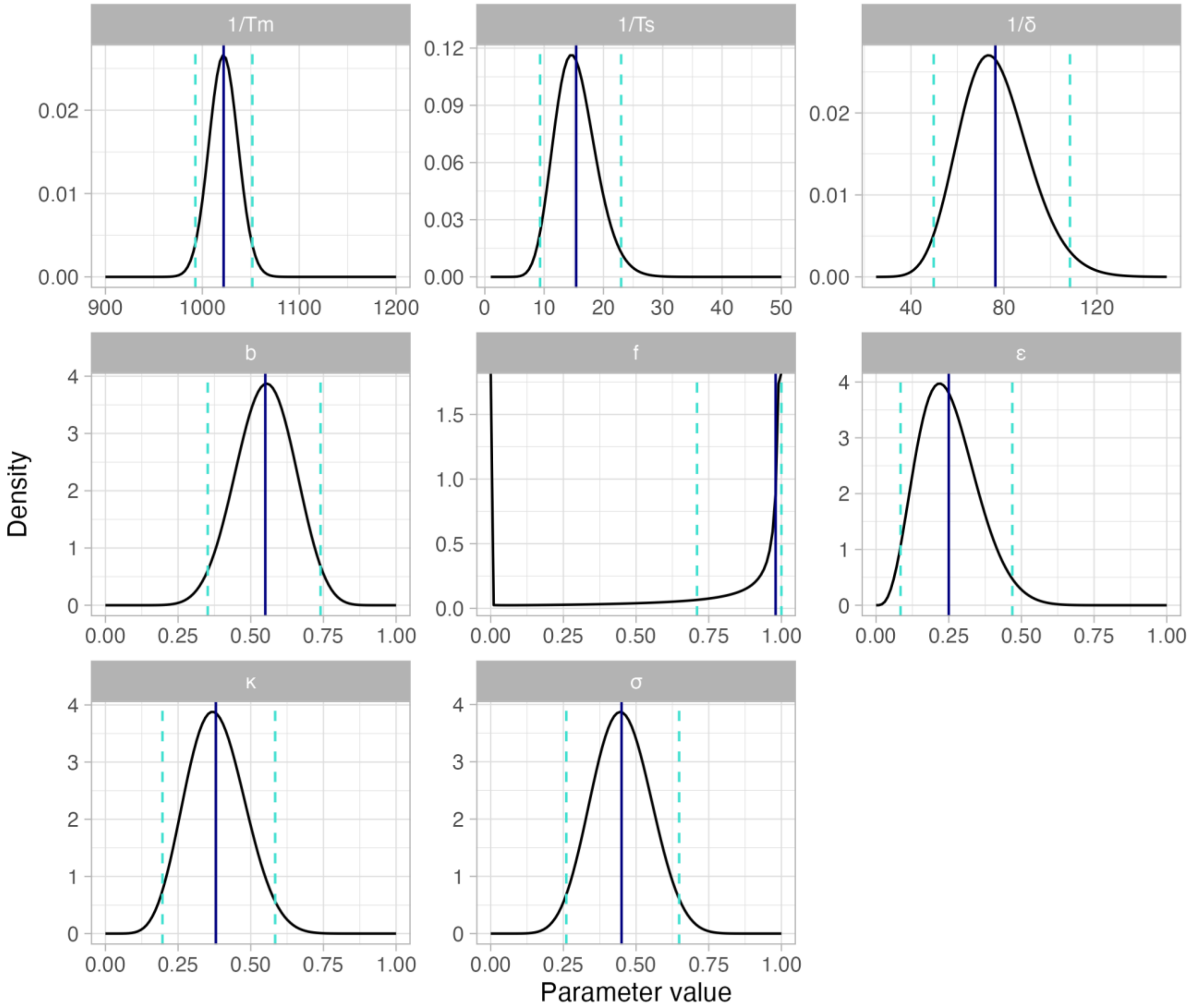

Supplementary Figure 2. Probability density functions for parameters included in global sensitivity analysis. Solid blue vertical lines depict the mean of the probability density function for each parameter, defined by the baseline value fitted using maximum likelihood estimation (MLE). Dashed teal vertical lines depict the lower and upper bounds of the 95% confidence interval for each parameter. For κ and f, since doxy-PEP was not incorporated in the calibration model, the mean value was estimated using the literature and expert opinion and assumption. Definitions of all parameters can be found in Table 1 of the main text. The 1,000 parameter sets for the global uncertainty analysis were generated from 1,000 random, independent draws for each distribution. Abbreviations: $1/T_m$, Average time to screen and treat, asymptomatic infection (days); $1/T_s$, Average time to screen and treat, symptomatic infection (days); 1/δ, Average time to natural recovery from infection (days); b, Transmission probability per partnership; f; Relative fitness of resistant bacteria, compared to susceptible; ε, Mixing parameter; κ, Proportion of doxy-PEP treatments that fail to prevent infection, for reasons not due to high-level tetracycline resistance; σ, Proportion of incident infections that are symptomatic.

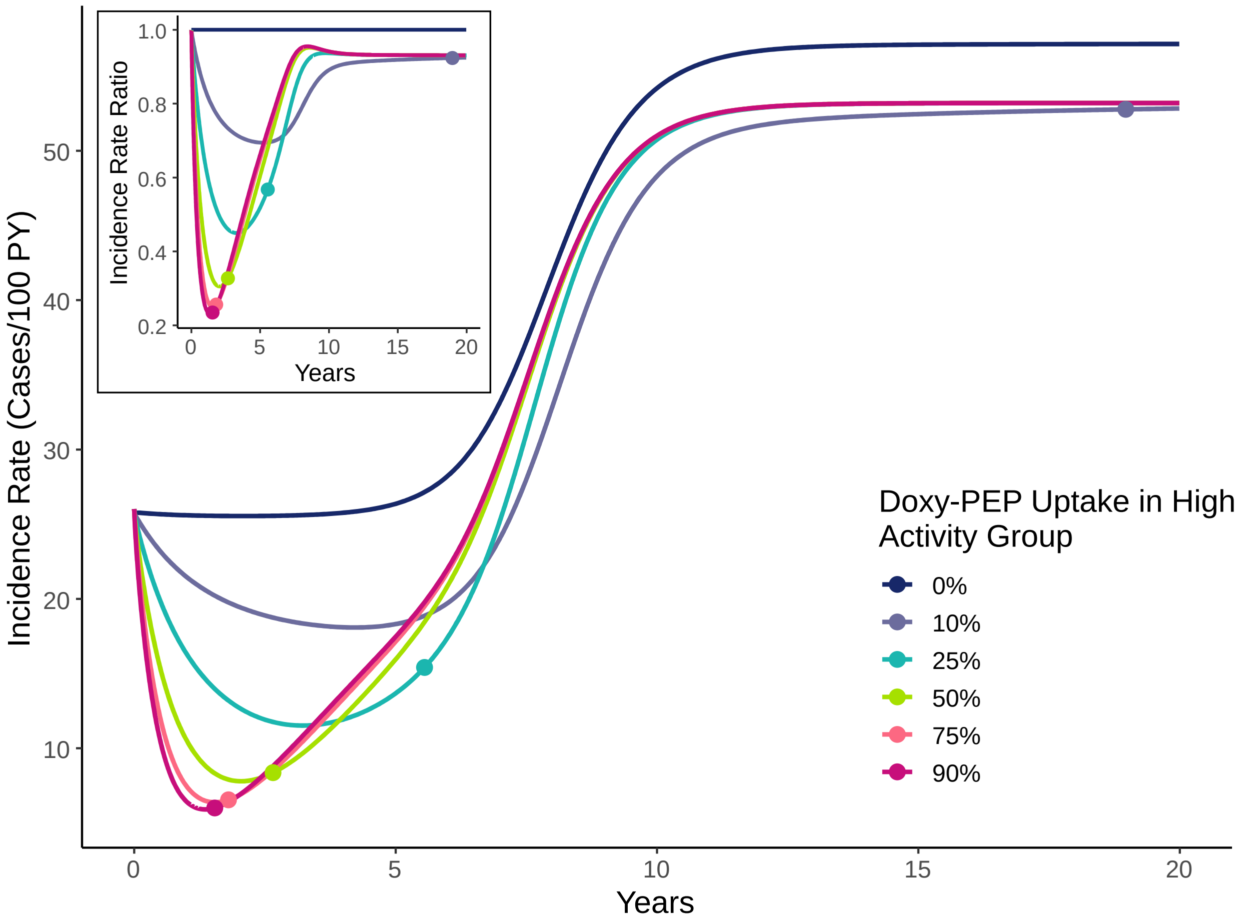

Supplementary Figure 3. Incidence rate of gonococcal infection over time for varying doxy-PEP uptake levels (%). The incidence rate is calculated as the number of incident cases per 100 years of susceptible person-time. The upper left inset visualizes the same data as an incidence rate ratio (IRR), where results are normalized (or divided by) the incidence rate under the no doxy-PEP introduction scenario (0% uptake). Lines are colored by doxy-PEP uptake, defined as the proportion of exposed individuals treated with doxy-PEP within the high sexual activity population. The points along these lines represent the time at which high-level tetracycline resistance reached an 84% prevalence among gonococcal infections under that uptake level, associated with loss of doxy-PEP's clinical utility. Abbreviations: doxy-PEP = doxycycline post-exposure prophylaxis, PY = person-years at risk.

Supplementary Table 1. Clinically relevant endpoints by level of doxy-PEP uptake (%) and prevalence of high-level tetracycline resistance at time 0. Median and IQRs are presented for 1000 simulations, with parameter values drawn randomly from their distributions for model parameters included in the global sensitivity analysis in Table 1 of the main text. The time until high-level tetracycline resistance at 84% prevalence is used to approximate time to loss of doxy-PEP's clinical utility, whereas the 5% prevalence threshold for ceftriaxone resistance indicates when new therapeutics would be needed. Dashes indicate that the result was not observed within the 20-year period over which the model was run.

	Initial prevalence of high-level tetracycline resistance	Doxy-PEP uptake (high sexual activity group only)					
		0%	10%	25%	50%	75%	90%
Time to key resistance thresholds (years), median (IQR)							
Ceftriaxone resistance, 5%	5%	4.9 (4.0-6.1)	5.0 (4.1-6.2)	4.9 (4.1-6.0)	4.7 (3.9-5.9)	4.7 (3.8-6.0)	4.6 (3.8-5.9)
	25%	4.9 (4.0-6.1)	4.9 (4.1-6.1)	4.8 (4.0-6.0)	4.8 (3.9-6.0)	4.8 (3.9-6.0)	4.8 (3.9-6.0)
	50%	4.9 (4.0-6.1)	4.9 (4.1-6.1)	4.9 (4.0-6.1)	4.9 (4.0-6.1)	4.8 (4.0-6.1)	4.8 (4.0-6.1)
	75%	4.8 (4.0-6.1)	4.9 (4.0-6.1)	4.9 (4.0-6.1)	4.9 (4.0-6.1)	4.9 (4.0-6.2)	4.9 (4.0-6.2)
Dual resistance, 5%	5%	-	6.3 (5.1-7.8)	5.1 (4.3-6.4)	4.7 (3.9-6.0)	4.7 (3.8-6.0)	4.6 (3.8-5.9)
	25%	6.3 (5.2-8.3)	5.4 (4.4-6.8)	5.0 (4.1-6.3)	4.8 (4.0-6.1)	4.8 (3.9-6.0)	4.8 (3.9-6.0)
	50%	5.6 (4.6-7.3)	5.1 (4.2-6.5)	4.9 (4.1-6.2)	4.9 (4.0-6.1)	4.9 (4.0-6.1)	4.8 (4.0-6.1)
	75%	5.2 (4.3-6.8)	5.0 (4.1-6.4)	4.9 (4.1-6.3)	4.9 (4.0-6.2)	4.9 (4.0-6.2)	4.9 (4.0-6.2)

High-level tetracycline resistance, 84%	5%	-	14.8 (12.0-19.4)	6.2 (5.1-7.4)	3.3 (2.7-4.0)	2.2 (1.8-2.7)	1.9 (1.6-2.3)
	25%	-	8.9 (7.3-11.2)	4.1 (3.4-4.9)	2.1 (1.7-2.5)	1.4 (1.2-1.7)	1.2 (1.0-1.5)
	50%	-	5.9 (4.9-7.2)	2.5 (2.1-3.1)	1.3 (1.1-1.6)	0.9 (0.8-1.1)	0.8 (0.7-1.0)
	75%	-	2.2 (1.8-2.7)	0.9 (0.8-1.1)	0.5 (0.4-0.6)	0.4 (0.3-0.4)	0.3 (0.3-0.4)
Percent reduction in 5-year outcomes compared to no doxy-PEP uptake, median (IQR)							
Prevalence of gonococcal infection	5%	0%	41.6% (13.8-63.7%)	63.9% (17.4-87.6%)	63.4% (5.3-92.3%)	59.3% (1.9-92.5%)	57.8% (1.2-92.4%)
	25%	0%	25.2% (7.2-49.1%)	29.0% (4.8-65.4%)	23.3% (0.9-67.6%)	19.9% (0.2-66.5%)	18.7% (0.1-65.9%)
	50%	0%	13.8% (3.4-31.8%)	13.9% (1.7-41.4%)	10.5% (0.3-41.2%)	8.2% (0.0-40.0%)	7.5% (0.0-39.4%)
	75%	0%	6.3% (1.3-15.5%)	5.4% (0.6-20.1%)	3.6% (0.1-19.1%)	3.1% (0.0-18.3%)	2.8% (0.0-17.9%)
Cumulative gonococcal infections	5%	0%	15.6% (9.0-24.1%)	32.7% (19.8-45.9%)	44.8% (28.5-60.5%)	51.5% (32.4-66.4%)	53.8% (33.5-68.7%)
	25%	0%	11.2% (6.5-17.5%)	21.1% (12.1-30.5%)	28.6% (15.6-39.2%)	32.4% (16.4-44.0%)	33.6% (16.6-46.3%)
	50%	0%	7.1% (3.9-11.1%)	12.5% (6.8-18.3%)	16.4% (7.9-23.7%)	18.3% (8.3-26.4%)	18.6% (8.3-27.6%)
	75%	0%	3.3% (1.8-5.2%)	5.7% (2.9-8.5%)	7.3% (3.3-11.0%)	8.1% (3.4-12.2%)	8.3% (3.4-12.6%)
Percent reduction in 20-year outcomes compared to no doxy-PEP uptake, median (IQR)							

Prevalence of gonococcal infection	5%	0%	0.9% (0.3-20.8%)	0.0% (0.0-27.7%)	0.0% (0.0-41.8%)	0.0% (0.0-43.7%)	0.0% (0.0-43.1%)
	25%	0%	0.2% (0.1-14.5%)	0.0% (0.0-18.7%)	0.0% (0.0-26.1%)	0.0% (0.0-25.8%)	0.0% (0.0-25.0%)
	50%	0%	0.1% (0.0-8.5%)	0.0% (0.0-15.5%)	0.0% (0.0-15.8%)	0.0% (0.0-15.8%)	0.0% (0.0-15.8%)
	75%	0%	0.0% (0.0-7.4%)	0.0% (0.0-11.5%)	0.0% (0.0-11.0%)	0.0% (0.0-11.0%)	0.0% (0.0-11.0%)
Cumulative gonococcal infections	5%	0%	8.0% (4.4-13.9%)	10.9% (5.3-21.2%)	11.4% (5.6-30.3%)	11.4% (5.8-35.1%)	11.4% (5.8-36.7%)
	25%	0%	4.3% (2.2-8.8%)	5.2% (2.4-14.9%)	5.4% (2.5-19.5%)	5.4% (2.6-24.3%)	5.4% (2.6-25.7%)
	50%	0%	2.5% (1.1-5.7%)	2.6% (1.2-9.4%)	2.7% (1.3-12.4%)	2.7% (1.3-14.7%)	2.7% (1.3-15.8%)
	75%	0%	1.0% (0.5-2.8%)	1.1% (0.5-4.5%)	1.1% (0.5-6.0%)	1.1% (0.5-7.3%)	1.1% (0.5-7.9%)

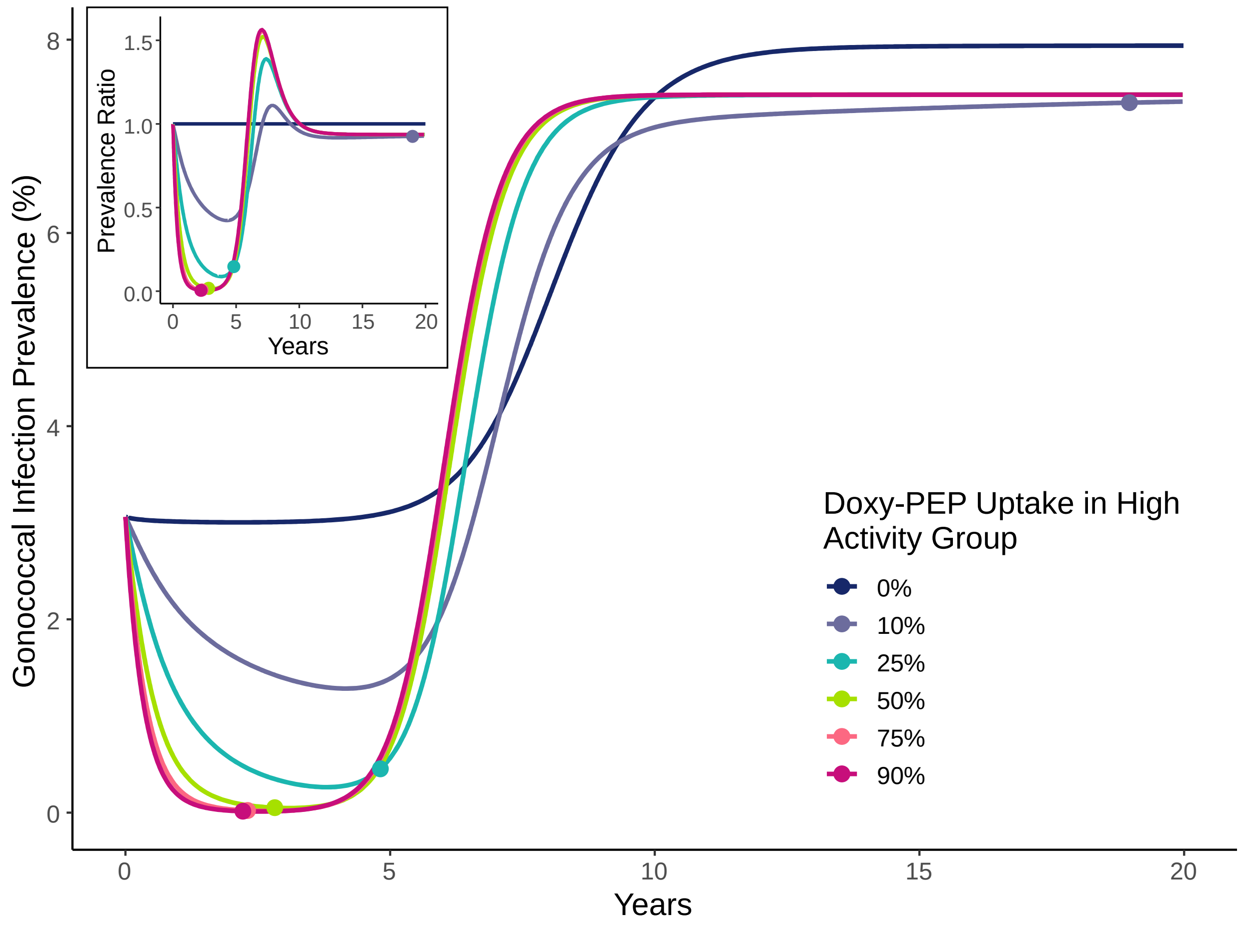

Supplementary Figure 4. Prevalence of gonococcal infection over time for varying doxy-PEP uptake levels (%) and STI screening rates. Prevalence at each timepoint is calculated as the total number of infections over the total population size ($N = 10^6$). The upper left inset visualizes the same data as a prevalence ratio (PR), where results are normalized (or divided by) the prevalence under the no doxy-PEP introduction scenario (0% uptake). Lines are colored by doxy-PEP uptake, defined as the proportion of exposed individuals treated with doxy-PEP within the high sexual activity population. The points along these lines represent the time at which high-level tetracycline resistance reached 84% prevalence under that uptake level, associated with loss of doxy-PEP's clinical utility. Each doxy-PEP uptake level is associated with a unique STI screening rate for gonococcal infection, calculated as a strictly increasing function of the doxy-PEP uptake level, that applies only to the doxy-PEP eligible high sexual activity group (see Technical Supplement, p. 15). The screening rate (T_m) was held constant at 0.36 in the low and intermediate sexual activity groups. Abbreviations: doxy-PEP = doxycycline post-exposure prophylaxis.

Supplementary Table 2. Clinically relevant endpoints by level of doxy-PEP uptake (%) and STI screening rate. Each doxy-PEP uptake level is associated with a unique STI screening rate for gonococcal infection, calculated as a strictly increasing function of the doxy-PEP uptake level, that applies only to the doxy-PEP eligible high sexual activity group (see Technical Supplement, p. 15). The screening rate (T_m) was held constant at 0.36 in the low and intermediate sexual activity groups. Median and IQRs are presented for 1000 simulations, with parameter values drawn randomly from their distributions for model parameters included in the global sensitivity analysis in Table 1 of the main text. The time until high-level tetracycline resistance at 84% prevalence is used to approximate time to loss of doxy-PEP's clinical utility, whereas the 5% prevalence threshold for ceftriaxone resistance indicates when new therapeutics would be needed. Dashes indicate that the result was not observed within the 20-year period over which the model was run. Negative values for percent reductions indicate that a percent increase was observed in the outcome compared to no doxy-PEP uptake.

	Doxy-PEP uptake (high sexual activity group only)					
	0%	10%	25%	50%	75%	90%
Screening rate per year in high sexual activity group ($T_{m,\ new}$)	0.36	0.72	1.27	2.18	3.09	3.64
Time to key resistance thresholds (years), median (IQR)						
Ceftriaxone resistance, 5%	4.9<br>(4.0-6.2)	4.0<br>(3.4-4.8)	3.2<br>(2.8-3.6)	2.4<br>(2.2-2.7)	2.0<br>(1.8-2.2)	1.9<br>(1.7-2.1)
Dual resistance, 5%	7.5<br>(6.0-10.1)	4.8<br>(4.1-5.8)	3.4<br>(3.0-3.9)	2.4<br>(2.2-2.8)	2.0<br>(1.8-2.3)	1.9<br>(1.7-2.1)
High-level tetracycline resistance, 84%	-	12.0<br>(9.7-15.6)	4.7<br>(4.0-5.6)	2.8<br>(2.3-3.3)	2.3<br>(1.9-2.8)	2.2<br>(1.8-2.7)
Maximum percent reduction achieved over 20 years compared to no doxy-PEP uptake, median (IQR)						
Prevalence of gonococcal infection	0%	65.2%<br>(32.7-87.6%)	92.2%<br>(69.6-97.9%)	98.4%<br>(92.0-99.5%)	99.3%<br>(97.2-99.8%)	99.6%<br>(98.3-99.8%)

Percent reduction in 5-year outcomes compared to no doxy-PEP uptake, median (IQR)						
Prevalence of gonococcal infection	0%	56.7% (16.7-84.5%)	79.5% (12.3-97.3%)	81.3% (-22.4-98.4%)	80.7% (-32.5-98.5%)	80.9% (-34.3-98.5%)
Cumulative gonococcal infections	0%	28.4% (17.7-40.2%)	51.4% (35.0-67.3%)	65.4% (46.7-80.6%)	72.0% (52.9-84.6%)	74.5% (54.5-86.0%)
Cumulative ceftriaxone treatments	0%	27.0% (16.9-38.6%)	49.7% (34.5-65.2%)	64.6% (49.8-79.3%)	70.5% (56.6-83.7%)	72.4% (59.3-85.3%)
Percent reduction in 20-year outcomes compared to no doxy-PEP uptake, median (IQR)						
Prevalence of gonococcal infection	0%	0.4% (0.1-23.2%)	0.0% (0.0-42.4%)	0.0% (0.0-51.1%)	0.0% (0.0-54.6%)	0.0% (0.0-54.4%)
Cumulative gonococcal infections	0%	8.0% (3.6-19.4%)	9.3% (4.6-32.5%)	9.6% (5.3-44.5%)	9.7% (5.4-51.9%)	9.7% (5.5-55.1%)
Cumulative ceftriaxone treatments	0%	11.9% (8.4-19.5%)	17.2% (12.4-31.5%)	21.1% (14.9-39.7%)	22.5% (15.9-44.1%)	23.0% (16.2-46.8%)

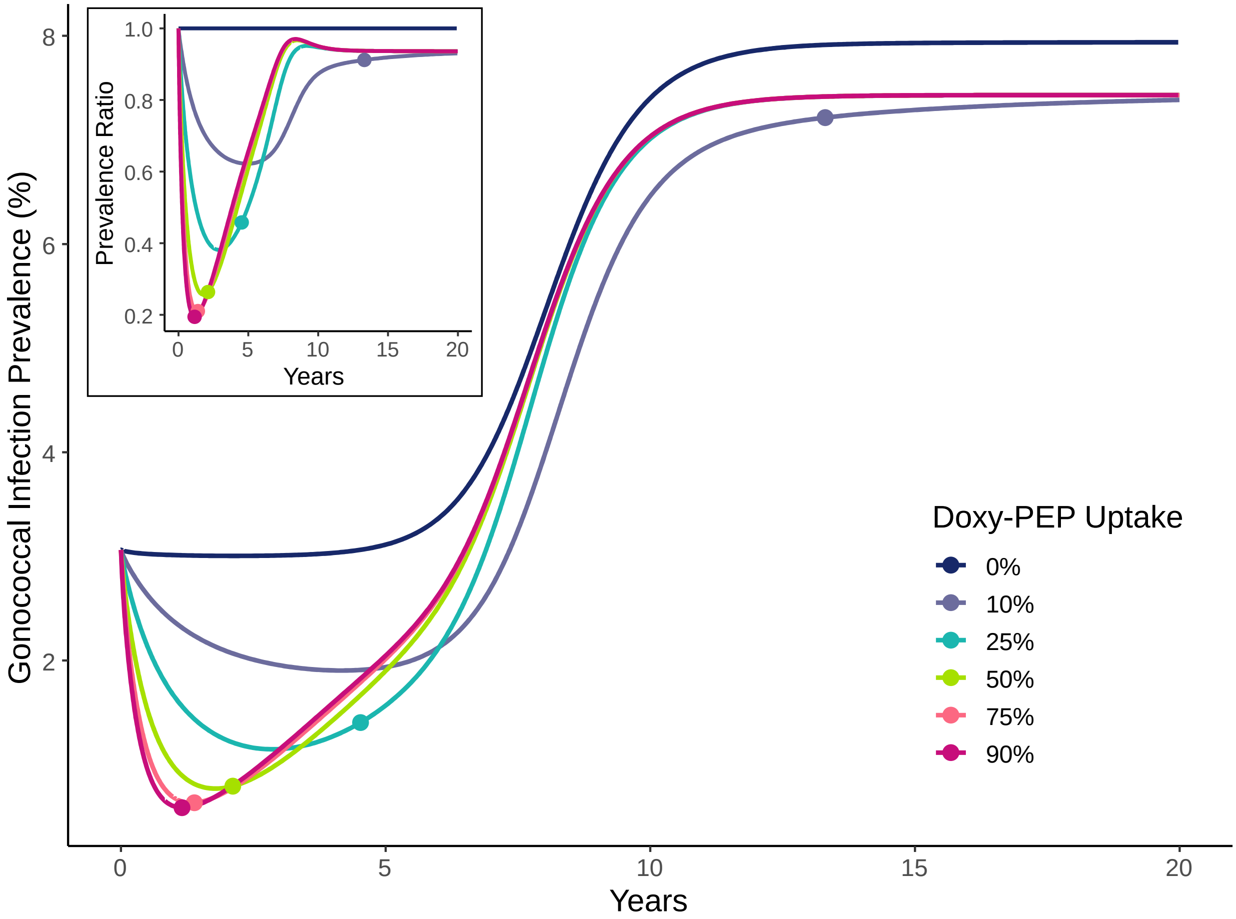

Supplementary Figure 5. Prevalence of gonococcal infection over time for varying doxy-PEP uptake levels (%) in the entire population. Doxy-PEP implementation is universal (i.e., not restricted to the high sexual activity group) and equivalent across sexual activity groups. Prevalence at each timepoint is calculated as the total number of infections over the total population size ($N = 10^6$). The upper left inset visualizes the same data as a prevalence ratio (PR), where results are normalized (or divided by) the prevalence under the no doxy-PEP introduction scenario (0% uptake). Lines are colored by doxy-PEP uptake, defined as the proportion of exposed individuals treated with doxy-PEP within the population. The points along these lines represent the time at which high-level tetracycline resistance reached 84% prevalence under that uptake level, associated with loss of doxy-PEP's clinical utility. Abbreviations: doxy-PEP = doxycycline post-exposure prophylaxis.

Supplementary Table 3. Clinically relevant endpoints by level of doxy-PEP uptake (%) in the entire population. Doxy-PEP implementation is universal (i.e., not restricted to the high sexual activity group) and equivalent across sexual activity groups. Median and IQRs are presented for 1000 simulations, with parameter values drawn randomly from their distributions for model parameters included in the global sensitivity analysis in Table 1 of the main text. The time until high-level tetracycline resistance at 84% prevalence is used to approximate time to loss of doxy-PEP's clinical utility, whereas the 5% prevalence threshold for ceftriaxone resistance indicates when new therapeutics would be needed. Dashes indicate that the result was not observed within the 20-year period over which the model was run.

	Doxy-PEP uptake (entire population)					
	0%	10%	25%	50%	75%	90%
Time to key resistance thresholds (years), median (IQR)						
Ceftriaxone resistance, 5%	4.9 (4.1-6.1)	4.9 (4.1-6.2)	4.8 (4.0-6.0)	4.7 (3.9-6.0)	4.7 (3.9-6.0)	4.7 (3.9-6.0)
Dual resistance, 5%	7.3 (6.0-9.9)	5.6 (4.6-7.0)	4.9 (4.0-6.2)	4.7 (3.9-6.0)	4.7 (3.9-6.0)	4.7 (3.9-6.0)
High-level tetracycline resistance, 84%	-	9.5 (8.0-11.9)	4.3 (3.7-5.3)	2.1 (1.8-2.6)	1.4 (1.2-1.7)	1.2 (1.0-1.4)
Maximum percent reduction achieved over 20 years compared to no doxy-PEP uptake, median (IQR)						
Prevalence of gonococcal infection	0%	45.5% (21.9-84.7%)	66.5% (45.5-85.1%)	75.8% (65.1-85.1%)	79.4% (72.8-85.0%)	80.8% (75.8-85.2%)
Percent reduction in 5-year outcomes compared to no doxy-PEP uptake, median (IQR)						
Prevalence of gonococcal infection	0%	41.4% (14.7-65.9%)	49.0% (9.6-82.9%)	42.3% (1.5-84.9%)	37.3% (0.4-84.1%)	36.0% (0.3-83.9%)
Cumulative gonococcal infections	0%	17.3% (10.6-26.0%)	31.4% (20.3-44.9%)	41.6% (25.1-55.6%)	47.4% (26.9-60.8%)	49.4% (27.5-63.0%)

Cumulative ceftriaxone treatments	0%	20.5% (14.5-28.7%)	37.5% (26.1-49.4%)	49.7% (30.4-60.9%)	56.7% (31.2-67.5%)	59.6% (31.1-70.4%)
Percent reduction in 20-year outcomes compared to no doxy-PEP uptake, median (IQR)						
Prevalence of gonococcal infection	0%	0.3% (0.1-20.3%)	0.0% (0.0-34.3%)	0.0% (0.0-33.6%)	0.0% (0.0-33.0%)	0.0% (0.0-32.6%)
Cumulative gonococcal infections	0%	7.0% (4.0-13.3%)	8.4% (4.3-21.3%)	8.6% (4.4-29.2%)	8.6% (4.4-34.5%)	8.6% (4.5-37.0%)
Cumulative ceftriaxone treatments	0%	10.0% (6.8-16.0%)	11.8% (7.5-27.4%)	12.3% (7.5-38.5%)	12.3% (7.4-46.2%)	12.3% (7.3-49.6%)

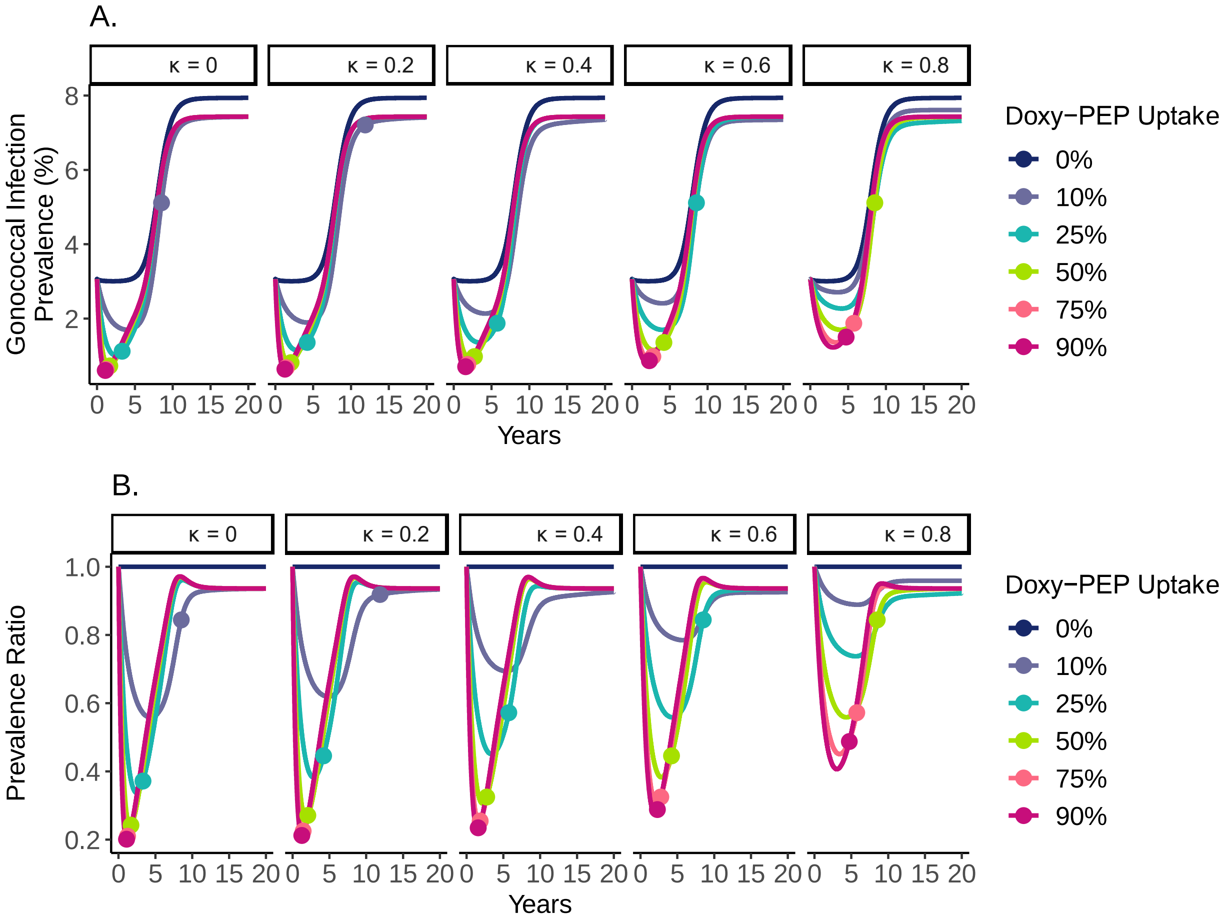

Supplementary Figure 6. Prevalence of gonococcal infection over time for varying doxy-PEP uptake levels (%), by the per exposure risk ratio of infection for doxy-PEP users relative to non-users exposed to doxy-PEP susceptible strains. A) Absolute prevalence estimates over time, calculated as the total number of gonococcal infections over the total population size ($N = 10^6$) under each doxy-PEP utilization scenario. **B)** Prevalence ratio (PR) estimates over time, where results are normalized (or divided by) the prevalence under the no doxy-PEP introduction scenario (0% uptake). Lines are colored by doxy-PEP uptake, defined as the proportion of exposed individuals treated with doxy-PEP within the high sexual activity population. The points along these lines represent the time at which high-level tetracycline resistance has reached an 84% prevalence among gonococcal infections under that uptake level, associated with loss of doxy-PEP's clinical utility. Vertical facets (columns) show the per exposure risk ratio of infection for doxy-PEP users relative to non-users exposed to doxy-PEP susceptible strains (tetracycline MIC ≤ 8 μg/mL), also explained as the proportion of doxy-PEP treatments that fail to prevent infection for reasons not due to high-level tetracycline resistance (κ). This is relative to the risk of infection for individuals exposed that are not using doxy-PEP. Under baseline model paramaterization, κ = 0.38. Abbreviations: doxy-PEP = doxycycline post-exposure prophylaxis, MIC = minimum inhibitory concentration.

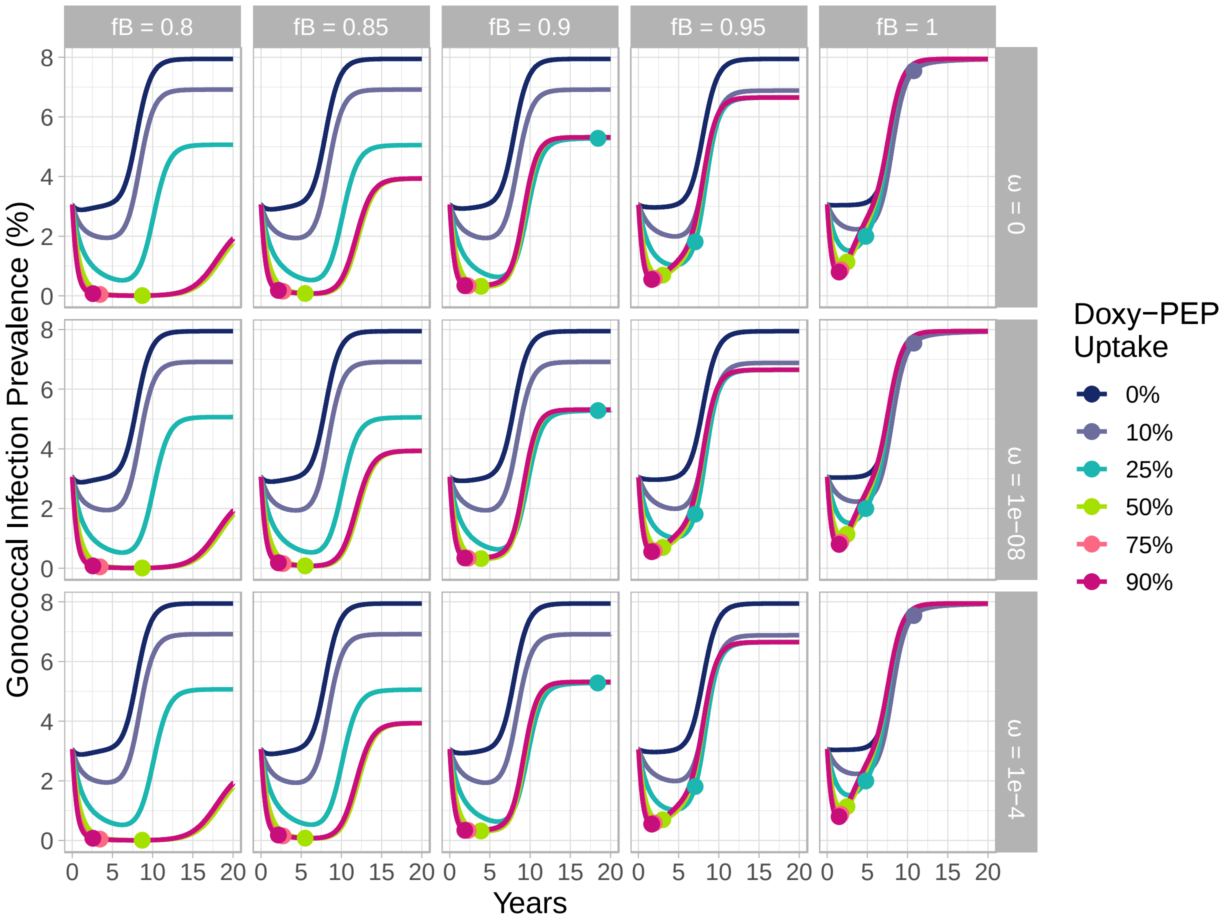

Supplementary Figure 7. Prevalence of gonococcal infection over time for varying doxy-PEP uptake levels (%), by properties of doxy-PEP. Lines are colored by doxy-PEP uptake, defined as the proportion of exposed individuals treated with doxy-PEP within the high sexual activity population. The points along these lines represent the time at which high-level tetracycline resistance has reached an 84% prevalence among gonococcal infections under that uptake level, associated with loss of doxy-PEP's clinical utility. Vertical facets (columns) show the relative fitness of high-level tetracycline resistant strains (MIC >8 µg/mL) compared to doxy-PEP susceptible strains with tetracycline MICs ≤ 8 µg/mL (f_B). Horizontal facets (rows) represent probabilities of *de novo* resistance emergence upon doxy-PEP use (ω_B). Under baseline model parameterization, f_B = 0.98 and ω_B = 0. Abbreviations: doxy-PEP = doxycycline post-exposure prophylaxis, MIC = minimum inhibitory concentration.

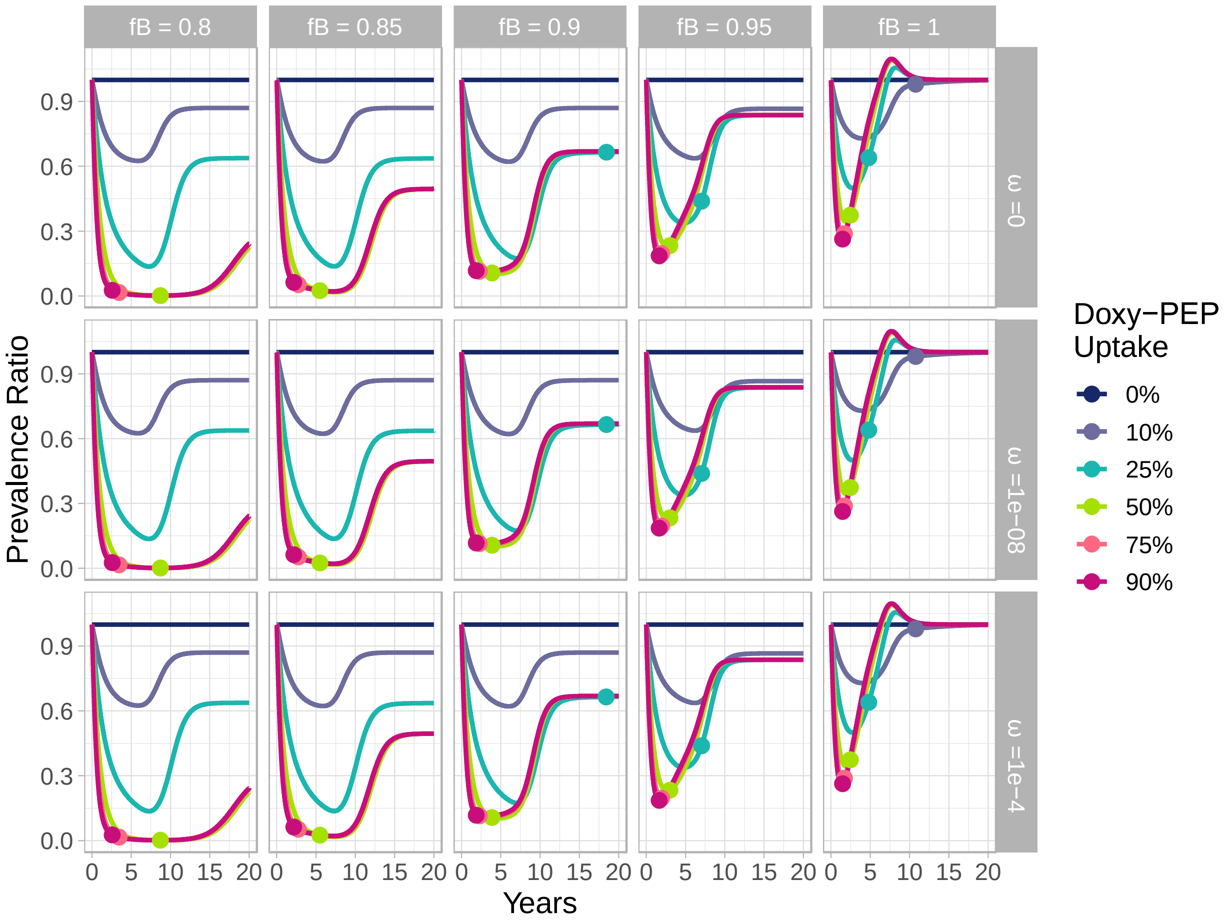

Supplementary Figure 8. Prevalence ratio of gonococcal infection over time for varying doxy-PEP uptake levels (%) relative to no doxy-PEP uptake, by properties of doxy-PEP. Lines are colored by doxy-PEP uptake, defined as the proportion of exposed individuals treated with doxy-PEP within the high sexual activity population. The prevalence ratio is calculated at each timepoint by dividing the prevalence of gonococcal infection under each doxy-PEP uptake scenario by the prevalence given 0% doxy-PEP uptake. The points along these lines represent the time at which high-level tetracycline resistance has reached an 84% prevalence among gonococcal infections under that uptake level, associated with loss of doxy-PEP's clinical utility. Vertical facets (columns) show the relative fitness of high-level tetracycline resistant strains (MIC >8 µg/mL) compared to doxy-PEP susceptible strains with tetracycline MICs ≤8 µg/mL (f_B). Horizontal facets (rows) represent probabilities of *de novo* resistance emergence upon doxy-PEP use (ω_B). Under baseline model parameterization, $f_B = 0.98$ and $\omega_B = 0$. Abbreviations: doxy-PEP = doxycycline post-exposure prophylaxis, MIC = minimum inhibitory concentration.

Technical Supplement

Model Structure

We adapted a single sex compartmental gonorrhea transmission model.[1] A visual overview of the susceptible-exposed-infectious-susceptible (SEIS) model is presented in Supplementary Figure 1. As previously described, infections are stratified by symptomatic (Y) versus asymptomatic (Z) status, as well as by resistance profile, where each infection can be caused by bacteria resistant to ceftriaxone (A), tetracycline (B), neither (0), or both (AB). The total size of the population (N) is set at 10^6 and the absolute size of each sexual activity group at N_k. Therefore,

$$N_k = S_k + E_k + I_k, \text{ where}$$
$$E_k = E_{0k} + E_{Ak} + E_{Bk} + E_{ABk}, \text{ and}$$
$$I_k = Y_{0k} + Y_{Ak} + Y_{Bk} + Y_{ABk} + Z_{0k} + Z_{Ak} + Z_{Bk} + Z_{ABk}$$

The relative rate of partner change (r_k) for each sexual activity group is drawn from estimates by Tuite et al.[2] determined by data from the National HIV Behavioral Surveillance System[3] ($r_1 = 1$, $r_2 = 5$, $r_3 = 20$). The minimum rate of partner change (c_{min}) was estimated through the maximum likelihood estimation (MLE) model fitting procedure. The annual rate of partner change for each activity group (θ_k) is therefore described by the equation:

$$\theta_k = r_k * c_{min}$$

Assortativity between sexual activity groups is characterized by mixing parameter ε [which can range between 0 (random mixing) and 1 (fully assortative mixing) and was determined through model fitting]. This leads the probability of an individual from sexual activity group i coming into sexual contact with an individual from group j to be:

$$p_{ij} = \varepsilon x_{ij} + (1-\varepsilon)\frac{\theta_j N_j}{\sum_{k=1}^{3} \theta_k N_k}$$

where x_{ij} is equal to 1 if $i = j$ and equal to 0 if $i \neq j$. The rate of infection for susceptible individuals in sexual activity group i from infected partners of group j ($\beta_{i\leftarrow j}$) is proportional to this per capita probability of sexual contact between groups i and j (p_{ij}), as well as the transmission probability per partnership (b). This relationship is defined by:

$$\beta_{i\leftarrow j} = bp_{ij}$$

Therefore, the per capita transmission matrix $\boldsymbol{\beta}$ is a $k \times k$ matrix with elements $\beta_{i\leftarrow j}$ at row i and column j, defined as the per capita rate of gonorrhea transmission to group i from group j. Note that the transmission matrix at element $\beta_{i\leftarrow j}$ is divided through by the size of group j's population,

which stays constant over time. As area is also held constant, and deemed not influential in per capita transmission rates, the model assumes constant density and yields results identical to frequency-dependent transmission.

Model Parameterization

We parameterized the model using the literature and the model fitting procedure, which used MLE to fit the model to a mean 3.0% baseline equilibrium prevalence of gonorrhea infection based on recent surveillance estimates in men who have sex with men (MSM) in the United States (U.S.).[4,5] The calibration SEIS model with ceftriaxone monotherapy and no doxy-PEP implementation was run over two years, and parameter MLEs were calculated using R package bbmle, which uses an optimizer function to find the minimum of the negative log-likelihood.[6] Specifically, the calibration model simulated *status quo* infection dynamics for two years, and at the end of the simulation (t=730 days) the final equilibrium gonorrhea prevalence was recorded. Assuming prevalence follows a beta distribution with variance $1.47 x 10^{-5}$, the model parameters that produced a 2-year predicted prevalence of gonococcal infection that made the observed surveillance estimate (3.0%) most probable were selected as the MLEs. Model parameters are listed in Table 1, and for parameters fit via MLE, starting values input to the fitting procedure are listed.

The model is calibrated using MLE to the single mean equilibrium prevalence target (3.0%) given that longitudinal data surveilling gonococcal prevalence in U.S. MSM is not available, and the ability for asymptomatic cases to go undetected makes prevalence difficult to estimate. The estimated cross-sectional, population-level prevalence of 3.0% used for model fitting does not reflect our uncertainty in the true prevalence of gonococcal infection in the U.S. MSM population. Estimating multiple parameters using a single prevalence target also means that individual model parameters are non-identifiable or cannot be obtained with precision. For these reasons, the MLEs for parameters included in the model fitting procedure are used only as 'baseline' scenarios. To reflect parameter uncertainty, all results are generated from 1000 simulated parameter sets, using random draws from the probability distributions for parameters that are not fixed (see Table 1; Supp. Figure 2). The baseline value for each parameter (i.e., the MLE) was assigned to be the mean of the probability distribution. All proportions were assumed to be beta distributed with a standard deviation of 0.10. Average time to event parameters were assumed to be gamma distributed, with a reasonable variance determined using the literature or expert opinion and assumption (Table 1). Quantitative model outcomes were summarized using medians and interquartile ranges (IQRs) across these 1000 simulations.

Model results, particularly those visualized using only baseline parameterization, should not be interpreted as absolute predictions, but rather as projections of relative gonococcal infection and resistance dynamics under a set of explicit model parameterizations. Model output is best

interpreted relatively, consistent with the presentation of prevalence ratios, incidence rate ratios, and percent differences.

The cross-sectional proportion of symptomatic infections and the proportion of infections distributed to each sexual activity group at the start of the model is set to the mean proportion achieved over the 2-year calibration SEIS model run with no doxy-PEP implementation. At t=0, the proportion of infections that are symptomatic is 13%. The prevalence of gonorrhea in the low, intermediate, and high sexual activity groups is 0.5%, 2.6%, and 13.7%, respectively, achieving a population-level weighted prevalence of 3.0%. Given the sizes of the low, intermediate, and high sexual activity groups ($N_1 = 3*10^5$, $N_2 = 6*10^5$, and $N_3 = 1*10^5$, respectively), 5% of gonococcal infections affect the low sexual activity group, 50% the intermediate group, and 45% the high group at the model start. The initial proportion of exposed individuals is also determined from the calibration model and set to 0.07% across all sexual activity groups at t=0.

Parameters describing treatment and retreatment rates by infection type (symptomatic vs. asymptomatic), as well as the rate of natural clearance of infection, are presented in Table 1. The treatment rate T_{sr} for those with an initial treatment failure assumes an average duration of infection three times that of those with initial treatment success; $1/T_{sr}$ represents the average time in days until successful retreatment, including the time it takes for: 1) the individual to receive the initial failed treatment, 2) the individual to re-seek care, and 3) the provider to identify and prescribe the correct antibiotic for retreatment. It is possible for resistance acquired upon initial treatment of a susceptible infection to then be retreated insufficiently with the same antibiotic before receiving a successful retreatment; in these instances, the average duration of infection is $(1/T_s + 1/T_{sr})$, in days.

Isolates from the 2018 Gonococcal Isolate Surveillance Project (GISP) were used to inform the proportion of tetracycline resistant infections. Given that only tetracycline, not doxycycline, resistance is reported, the prevalence of high-level (MIC >8 µg/mL) tetracycline resistance in MSM (0.109, or 10.9%) was used as a proxy measure to define the proportion of doxy-PEP resistant infections.[7] While interpretative breakpoints for *N. gonorrhoeae* susceptibility and resistance to doxycycline have not been defined, treatment failures have been observed when isolates have doxycycline MICs ≥1 µg/ml,[8] and doxycycline MICs correlate with tetracycline MICs.[9] Moreover, a trial of 200mg of minocycline (another tetracycline class antibiotic) as post-exposure prophylaxis for gonorrhea in men with urethritis did not prevent infection when isolates had tetracycline MICs >2 µg/mL.[10] We conservatively assume that only high-level tetracycline resistance (MIC >8 µg/mL) renders doxy-PEP ineffective and that strains with lower MICs, even those classified as resistant (2-8 µg/mL), are doxy-PEP susceptible given that therapeutic and prophylactic efficacies might not align. To account for uncertainty and to allow generalizability of results to other geographic settings, we vary this initial prevalence of resistance in sensitivity

analysis (0.05-0.75). Estimates from 2020-2021 GISP data in MSM were used to inform the starting prevalence of ceftriaxone resistance (0.01%).[11]

Parameters of ceftriaxone, including the probability of *de novo* resistance (ω_A) and the fitness cost associated with resistance ($1-f_A$), were inferred from the literature.[2,12] In our primary analysis, high-level tetracycline resistance (MIC >8 μg/mL), equated to doxy-PEP resistance, was assumed to confer the same small fitness cost (2%) as ceftriaxone resistance relative to all other strains (MIC ≤8 μg/mL); sensitivity analyses explored outcomes over a wider range of potential fitness costs ($1-f_B$: 0-20%). As resistance is treated as a binary trait, the *de novo* resistance parameter ω_B characterizes the probability of an infection transitioning from a susceptible to resistant state upon doxy-PEP use. We assumed resistance emergence upon doxy-PEP use was 0% at baseline, but this also varied in sensitivity analyses (ω_B: $0\text{-}10^{-4}$). Parameters are only informed estimates of these drugs' probabilities of *de novo* resistance and resistance-associated fitness costs, which are inherently difficult properties to observe and measure *in vivo*. Ceftriaxone monotherapy was assumed to be the treatment given to 100% of those who progressed to gonococcal infection; those with symptomatic ceftriaxone resistant infections (for which this therapy is ineffective) could seek retreatment at rate T_{sr} with probability π_s. For these cases, we assigned retreatment with a last-resort antibiotic external to our model for which we assumed complete efficacy and did not monitor resistance trends.

Doxy-PEP therapy was assumed to be completely unsuccessful in preventing infection caused by exposure to a high-level tetracycline resistant strain. 'Exposure' is not represented at the sexual contact level but rather at the partnership level and is defined by a gonococcal infection transmission event to a susceptible individual. Data from the U.S. DoxyPEP trial showed a 55% reduced quarterly cumulative incidence of gonococcal infection for MSM and transgender women doxy-PEP users (RR = 0.45) compared to non-users.[13] Therefore, assuming that 10.9% of those receiving doxy-PEP had risk ratio [RR] = 1 due to pre-existing high-level tetracycline resistance, we used a weighted average to calculate the RR for individuals exposed to strains with lower MICs (≤ 8 μg/mL) that would allow us to observe an overall RR = 0.45.

$$(RR = 0.45) = 0.109 * (RR = 1) + 0.891 * \kappa$$

This implied the quarterly relative risk of infection for the remaining 89.1% of exposures was $\kappa = 0.38$ for doxy-PEP users relative to non-users, consistent with the range of efficacies in preventing any STI, including chlamydia and syphilis, reported for the DoxyPEP trial.[13] For the primary analysis, we made the simplifying and optimistic assumption that this corresponded to a 62% reduction in the risk of infection *per exposure* (i.e., the proportion of doxy-PEP treatments that failed to prevent gonococcal infection for reasons not due to high-level resistance, including but not limited to medication nonadherence, was $\kappa = 0.38$). This holds if individuals in the DoxyPEP study had *N. gonorrhoeae* exposure once on average during a three-month period. To

test the impact of this assumption, we explored a range of risk ratios per exposure (κ: 0-0.8) in sensitivity analyses.

Doxy-PEP uptake is defined by the proportion of exposed individuals at a given time point who use doxy-PEP, rather than the proportion of individuals who will use doxy-PEP upon exposure. Therefore, a 50% uptake could imply that within the high activity group, 50% of individuals accepted and used doxy-PEP for 100% of exposures, or that 100% of individuals accepted and used doxy-PEP but for only 50% of exposures – for model purposes, results are equivalent.

Model Equations

In matrix form, the model is described by the following system of differential equations, where ∘ denotes element-wise multiplication. All parameters are defined in Table 1. Bolded letters represent $k \times 1$ column vectors with compartmental variables for each sexual activity group (e.g., $\mathbf{S} = [S_1,...S_k]^T$) for k groups), with the exception of the $k \times k$ matrix $\boldsymbol{\beta}$.

$$d\mathbf{S}/dt = -\boldsymbol{\beta}((\mathbf{Y}_0 + \mathbf{Z}_0) + f_A(\mathbf{Y}_A + \mathbf{Z}_A) + f_B(\mathbf{Y}_B + \mathbf{Z}_B) + f_{AB}(\mathbf{Y}_{AB} + \mathbf{Z}_{AB}))\circ\mathbf{S} +$$
$$(1-\omega_A)(T_s\mathbf{Y}_0 + T_m\mathbf{Z}_0) +$$
$$\pi_s T_{sr}(\mathbf{Y}_A + \mathbf{Y}_{AB}) +$$
$$(1-\omega_A)(T_s\mathbf{Y}_B + T_m\mathbf{Z}_B) +$$
$$\xi_B(1-\kappa)(1-\omega_B)\gamma(\mathbf{E}_0 + \mathbf{E}_A) +$$
$$\delta(\mathbf{Y}_0 + \mathbf{Y}_A + \mathbf{Y}_B + \mathbf{Y}_{AB} + \mathbf{Z}_0 + \mathbf{Z}_A + \mathbf{Z}_B + \mathbf{Z}_{AB}) +$$
$$\rho\mathbf{N} - \rho\mathbf{S}$$

$$d\mathbf{E}_0/dt = \boldsymbol{\beta}(\mathbf{Y}_0 + \mathbf{Z}_0)\circ\mathbf{S} - \gamma\mathbf{E}_0$$
$$d\mathbf{Y}_0/dt = \sigma(1-\xi_B)\gamma\mathbf{E}_0 + \sigma\xi_B\kappa(1-\omega_B)\gamma\mathbf{E}_0 - T_s\mathbf{Y}_0 - \delta\mathbf{Y}_0 - \rho\mathbf{Y}_0$$
$$d\mathbf{Z}_0/dt = (1-\sigma)(1-\xi_B)\gamma\mathbf{E}_0 + (1-\sigma)\xi_B\kappa(1-\omega_B)\gamma\mathbf{E}_0 - T_m\mathbf{Z}_0 - \delta\mathbf{Z}_0 - \rho\mathbf{Z}_0$$

$$d\mathbf{E}_A/dt = f_A\boldsymbol{\beta}(\mathbf{Y}_A + \mathbf{Z}_A)\circ\mathbf{S} - \gamma\mathbf{E}_A$$
$$d\mathbf{Y}_A/dt = \sigma(1-\xi_B)\gamma\mathbf{E}_A + \sigma\xi_B\kappa(1-\omega_B)\gamma\mathbf{E}_A + \omega_A T_s\mathbf{Y}_0 + \omega_A T_s\mathbf{Y}_0 - \pi_s T_{sr}\mathbf{Y}_A - \delta\mathbf{Y}_A - \rho\mathbf{Y}_A$$
$$d\mathbf{Z}_A/dt = (1-\sigma)(1-\xi_B)\gamma\mathbf{E}_A + (1-\sigma)\xi_B\kappa(1-\omega_B)\gamma\mathbf{E}_A + \omega_A T_m\mathbf{Z}_0 - \delta\mathbf{Z}_A - \rho\mathbf{Z}_A$$

$$d\mathbf{E}_B/dt = f_B\boldsymbol{\beta}(\mathbf{Y}_B + \mathbf{Z}_B)\circ\mathbf{S} - \gamma\mathbf{E}_B$$
$$d\mathbf{Y}_B/dt = \sigma\gamma\mathbf{E}_B + \sigma\xi_B\omega_B\gamma\mathbf{E}_0 - T_s\mathbf{Y}_B - \delta\mathbf{Y}_B - \rho\mathbf{Y}_B$$
$$d\mathbf{Z}_B/dt = (1-\sigma)\gamma\mathbf{E}_B + (1-\sigma)\xi_B\omega_B\gamma\mathbf{E}_0 - T_m\mathbf{Z}_B - \delta\mathbf{Z}_B - \rho\mathbf{Z}_B$$

$$d\mathbf{E}_{AB}/dt = f_{AB}\boldsymbol{\beta}(\mathbf{Y}_{AB} + \mathbf{Z}_{AB})\circ\mathbf{S} - \gamma\mathbf{E}_{AB}$$
$$d\mathbf{Y}_{AB}/dt = \sigma\gamma\mathbf{E}_{AB} + \sigma\xi_B\omega_B\gamma\mathbf{E}_A + \omega_A T_s\mathbf{Y}_B - \pi_s T_{sr}\mathbf{Y}_{AB} - \delta\mathbf{Y}_{AB} - \rho\mathbf{Y}_{AB}$$
$$d\mathbf{Z}_{AB}/dt = (1-\sigma)\gamma\mathbf{E}_{AB} + (1-\sigma)\xi_B\omega_B\gamma\mathbf{E}_A + \omega_A T_m\mathbf{Z}_B - \delta\mathbf{Z}_{AB} - \rho\mathbf{Z}_{AB}$$

We calculate the number of incident infections at time *t*, or the overall force of infection across sexual activity groups, as $\lambda_t = \boldsymbol{\beta}((\boldsymbol{Y}_{0t} + \boldsymbol{Z}_{0t}) + f_A(\boldsymbol{Y}_{At} + \boldsymbol{Z}_{At}) + f_B(\boldsymbol{Y}_{Bt} + \boldsymbol{Z}_{Bt}) + f_{AB}(\boldsymbol{Y}_{ABt} + \boldsymbol{Z}_{ABt}))\circ\boldsymbol{S}_t$.

The overall prevalence of infection at time t can be calculated as $\text{Prev}_t = (\boldsymbol{Y}_{0t} + \boldsymbol{Z}_{0t} + \boldsymbol{Y}_{At} + \boldsymbol{Z}_{At} + \boldsymbol{Y}_{Bt} + \boldsymbol{Z}_{Bt} + \boldsymbol{Y}_{ABt} + \boldsymbol{Z}_{ABt})/N$.

Tetracycline Resistance Threshold

As the estimated prevalence of high-level tetracycline in the U.S. MSM population exceeds 5% (i.e., the World Health Organization resistance benchmark for warranting new treatment guidelines) at baseline, we assume that a ≤10% reduction in risk of infection with doxy-PEP use would render it a non-useful preventive therapy for patients. Therefore, assuming those receiving doxy-PEP with pre-existing high-level tetracycline resistance had risk ratio [RR] = 1, and those without this resistance had RR $\kappa = 0.38$, we used a weighted average to calculate the prevalence of high-level resistance (p_R) that would allow us to observe an overall RR = 0.90. This results in a resistance prevalence threshold of interest of $p_R = 0.84$, or 84%.

$$(RR = 0.90) = p_R + (1 - p_R)\kappa$$

Sensitivity Analyses

Beyond sensitivity analyses exploring the impact of individual parameters, we conducted two additional analyses reflecting different doxy-PEP rollout strategies for comparison. The first kept doxy-PEP utilization restricted to the high sexual activity group but complemented this with accelerated STI screening for gonococcal infection. The screening rate (T_m) was held constant at 0.36 in the low and intermediate sexual activity groups but increased in the high activity group as a strictly increasing function of doxy-PEP uptake. Since we define doxy-PEP uptake as a proportion of all exposed individuals in the high sexual activity group, we also update the screening rate for the entire high sexual activity group using the function below, which averages i) an accelerated screening rate of 4.0 per year and ii) the baseline screening rate of 0.36 per year (T_m), weighted by the doxy-PEP uptake level (ξ_B) :

$$T_{m,new} = 4.0\,\xi_B + T_m(1 - \xi_B)$$

The second analysis expanded doxy-PEP utilization to all sexual activity groups, in line with a hypothetical policy making doxy-PEP available broadly to MSM without targeting individuals at high-risk. Under this approach, doxy-PEP uptake was equivalent for all individuals, irrespective of sexual activity group. We again explored a range of doxy-PEP use (0-90%) to facilitate comparison with the primary analysis, even though uptake as high as 90% may be unrealistic for

an entire population. As these uptake levels characterize the proportion of gonorrhea-exposed individuals receiving doxy-PEP among the entire population and not just a 10% subset, absolute use of doxy-PEP was increased.

References

1. Reichert E, Yaesoubi R, Rönn MM, Gift TL, Salomon JA, Grad YH. Resistance-minimising strategies for introducing a novel antibiotic for gonorrhoea treatment: a mathematical modelling study. *Lancet Microbe*. Published online August 21, 2023. doi:10.1016/S2666-5247(23)00145-3
2. Tuite AR, Gift TL, Chesson HW, Hsu K, Salomon JA, Grad YH. Impact of Rapid Susceptibility Testing and Antibiotic Selection Strategy on the Emergence and Spread of Antibiotic Resistance in Gonorrhea. *J Infect Dis*. 2017;216(9):1141-1149. doi:10.1093/infdis/jix450
3. Centers for Disease Control and Prevention. HIV Infection Risk, Prevention, and Testing Behaviors among Men Who Have Sex With Men -- National HIV Behavioral Surveillance, 20 U.S. Cities, 2014. HIV Surveillance Special Report 15. Published January 2016. Accessed September 8, 2022. http://www.cdc.gov/hiv/library/reports/surveillance/#panel2
4. Johnson Jones ML, Chapin-Bardales J, Bizune D, Papp JR, Phillips C, Kirkcaldy RD, et al. Extragenital Chlamydia and Gonorrhea Among Community Venue-Attending Men Who Have Sex with Men - Five Cities, United States, 2017. MMWR Morb Mortal Wkly Rep. 2019 Apr 12;68(14):321–5.
5. Grov C, Cain D, Rendina HJ, Ventuneac A, Parsons JT. Characteristics Associated With Urethral and Rectal Gonorrhea and Chlamydia Diagnoses in a US National Sample of Gay and Bisexual Men: Results From the One Thousand Strong Panel. Sex Transm Dis. 2016 Mar;43(3):165–71.
6. Bolker B, R Development Core Team. bbmle: Tools for General Maximum Likelihood Estimation [Internet]. 2022. Available from: https://cran.r-project.org/package=bbmle
7. Mortimer TD, Grad YH. A Genomic Perspective on the Near-term Impact of Doxycycline Post-exposure Prophylaxis on Neisseria gonorrhoeae Antimicrobial Resistance. *Clin Infect Dis*. Published online May 4, 2023:ciad279. doi:10.1093/cid/ciad279
8. Wiesner PJ, Holmes KK, Sparling PF, Maness MJ, Bear DM, Gutman LT, et al. Single doses of methacycline and doxycycline for gonorrhea: a cooperative study of the frequency and cause of treatment failure. J Infect Dis. 1973 Apr;127(4):461–6.
9. Whittington WL, Roberts MC, Hale J, Holmes KK. Susceptibilities of Neisseria gonorrhoeae to the glycylcyclines. Antimicrob Agents Chemother. 1995 Aug;39(8):1864–5.
10. Harrison WO, Hooper RR, Wiesner PJ, Campbell AF, Karney WW, Reynolds GH, et al. A Trial of Minocycline Given after Exposure to Prevent Gonorrhea. N Engl J Med. 1979 May 10;300(19):1074–8.
11. CDC. cdc.gov. 2022 [cited 2023 Mar 27]. Preliminary 2021 STD Surveillance Data. Available from: https://www.cdc.gov/std/statistics/2021/default.htm
12. Vegvari C, Grad YH, White PJ, Didelot X, Whittles LK, Scangarella-Oman NE, et al. Using rapid point-of-care tests to inform antibiotic choice to mitigate drug resistance in gonorrhoea. Eurosurveillance. 2020 Oct 29;25(43):1900210.
13. Luetkemeyer AF, Donnell D, Dombrowski JC, Cohen S, Grabow C, Brown CE, et al. Postexposure Doxycycline to Prevent Bacterial Sexually Transmitted Infections. N Engl J

Med. 2023 Apr 6;388(14):1296–306.
